# Integrated Epigenome, Exome, and Transcriptome Analyses Reveal Molecular Subtypes and Homeotic Transformation in Uterine Fibroids

**DOI:** 10.1016/j.celrep.2019.11.077

**Published:** 2019-12-17

**Authors:** Jitu Wilson George, Huihui Fan, Benjamin Johnson, Tyler James Carpenter, Kelly Katherine Foy, Anindita Chatterjee, Amanda Lynn Patterson, Julie Koeman, Marie Adams, Zachary Brian Madaj, David Chesla, Erica Elizabeth Marsh, Timothy Junius Triche, Hui Shen, Jose Manuel Teixeira

**Affiliations:** 1 Department of Obstetrics, Gynecology, and Reproductive Biology, College of Human Medicine, Michigan State University, Grand Rapids, MI, USA; 2 Center for Epigenetics, Van Andel Research Institute, Grand Rapids, MI, USA; 3 Bioinformatics and Biostatistics Core, Van Andel Research Institute, Grand Rapids, MI, USA; 4 Genomics Core, Van Andel Research Institute, Grand Rapids, MI, USA; 5 Spectrum Health Universal Biorepository, Spectrum Health System, Grand Rapids, MI, USA; 6 Division of Reproductive Endocrinology and Infertility, Department of Obstetrics and Gynecology, University of Michigan Medical School, Ann Arbor, MI, USA; 7 Division of Animal Sciences, Department of Obstetrics, Gynecology and Women’s Health, University of Missouri, Columbia, MO, USA; 8 These authors contributed equally; 9 Lead Contact

## Abstract

Uterine fibroids are benign myometrial smooth muscle tumors of unknown etiology that, when symptomatic, are the most common indication for hysterectomy in the United States. Unsupervised clustering of results from DNA methylation analyses segregates normal myometrium from fibroids and further segregates the fibroids into subtypes characterized by *MED12* mutation or activation of either *HMGA2* or *HMGA1* expression. Upregulation of *HMGA2* expression does not always appear to be dependent on translocation but is associated with hypomethylation in the *HMGA2* gene body. *HOXA13* expression is upregulated in fibroids and correlates with expression of typical uterine fibroid genes. Significant overlap of differentially expressed genes is observed between cervical stroma and uterine fibroids compared with normal myometrium. These analyses show a possible role of DNA methylation in fibroid biology and suggest that homeotic transformation of myometrial cells to a more cervical stroma phenotype could be an important mechanism for etiology of the disease.

## In Brief

George et al. detect DNA hypomethylation in the *HMGA2* gene body in uterine fibroids expressing high levels of *HMGA2*, regardless of translocation, suggesting an alternative mechanism of activation. They also observe *HOXA13* overexpression in fibroids, evidence of pathogenic homeotic transformation of myometrial cells to a more cervical stroma phenotype.

## Graphical Abstract

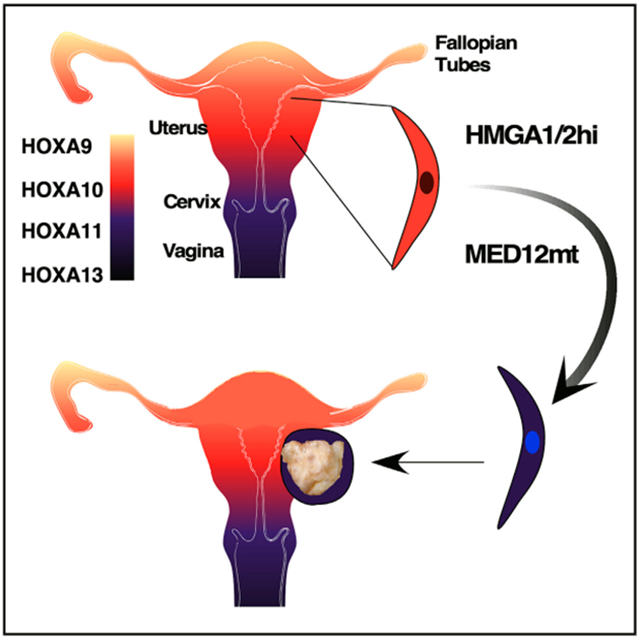

## INTRODUCTION

Uterine fibroids (also known as leiomyomas) are benign tumors that develop in the smooth muscle of the uterine myometrium (Figure S1A) and are estimated to occur in up to 75% of reproductive-age women. Although mostly asymptomatic, approximately 25% of women with fibroids suffer from clinically significant symptoms, including pelvic discomfort, menstrual bleeding, menorrhagia to preterm labor, recurrent pregnancy loss, and infertility (Bulun, 2013; Commandeur et al., 2015). There is a strong racial disparity in the disease, with a lifetime prevalence estimated to be 3 times higher in women of African descent (Jacoby et al., 2010), who also have earlier onset, a higher tumor burden, and greater severity of symptoms. Non-surgical, hormone-based therapies for fibroids offer only short-term mitigation of symptoms, and their use is limited because of significant associated side effects. Surgical intervention is often the last resort for women seeking permanent relief from the disease, and symptomatic fibroids are the most common indication for hysterectomy in the United States (Wu et al., 2007).

Approximately 30%–40% of fibroids have been reported as having karyotypic abnormalities, with the most commonly reported being translocations at chromosome regions of 12q15 and 6q21, leading to overexpression of the high mobility group AT-hook genes, *HMGA2* and *HMGA1*, respectively (Nilbert et al., 1990; Sandberg, 2005). HMGA family proteins are non-histone, chromatin-binding proteins that regulate transcription by influencing DNA conformation and, in the process, accessibility of DNA-binding proteins. Because of their various DNA-binding properties, they influence diverse cellular processes, including cell growth, proliferation, and cell death (Chen et al., 2010; Zhou et al., 1996).

More recently, whole-exome sequencing (WES) has identified somatic mutations in Mediator complex subunit 12 (*MED12*), most often in exon 2, that occur in around 50%–70% of fibroids (Mäkinen et al., 2011). *MED12* is located on the X chromosome and encodes a highly conserved 250-kDa protein that forms part of the Mediator RNA polymerase II pre-initiation complex. Together, *MED12* mutation (*MED12*mt) and *HMGA1* or *HMGA2* overexpression (*HMGA1*hi and *HMGA2*hi, respectively) encompass approximately 80%–90% of genetic alterations present in all fibroids (Bertsch et al., 2014; Mehine et al., 2014). However, the precise mechanisms disrupted during fibroid development or progression have yet to be determined.

Subtype classification of fibroids based on their mutation status or gene expression characteristics have been proposed (Mehine et al., 2016), but the DNA methylation profiles of these fibroid subtypes have not been reported. Additionally, in some cases, the subtyping was performed without consideration of fibroids from African-American women (Mehine et al., 2016). Methylation of cytosine nucleotides 5′ to a guanine (CpG) in DNA is among the most well-studied epigenetic marks known to influence gene expression (Jones, 2012), but how these epigenetic modifications might affect transcriptional activity in fibroids has not been well described, nor has cytosine methylation in a non-CpG context (CpH methylation) (Lister et al., 2009, 2013). The major goal of this study was to delineate the molecular landscape of fibroids based on integrated genome-wide analysis of DNA methylation and mRNA transcription in the context of their mutational status for subtype categorization and identify possible targetable mechanisms for therapeutic intervention.

## RESULTS

### Fibroid Subtype Determination

We applied an integrated approach to study uterine fibroid subtypes by combining DNA methylation array hybridization, WES, and RNA sequencing (RNA-seq) to determine driver mechanisms underlying subtype determination. Ten normal myometrial samples (5 Caucasian and 5 African-American) and 24 fibroid samples (12 Caucasian and 12 African-American) were collected for DNA methylation analyses. Methylomes for normal myometria and fibroids were profiled using the Infinium MethylationEPIC array (EPIC) (Zhou et al., 2017). Epidemiological studies have documented a strong racial disparity in the disease, with African-American women presenting with greater incidence, age of onset, and severity (Stewart et al., 2013). Self-identified race was confirmed using EPIC SNP probes (Figures S1B and S1C) with a published model (Zhou et al., 2017) to mitigate confounding effects from possible misidentification. To assess the cellular composition of the samples, promoter methylation of MIR200C/141, which are methylated in mesenchymal cells (Mongroo and Rustgi, 2010) but unmethylated in epithelial cells, was analyzed (Figure S1D). The methylation beta values, corresponding to the fraction of methylated probe signals, suggested very low contamination of the normal myometria with epithelial cells and approximately 80%–90% smooth muscle cells in the fibroids. Further examination of alpha smooth muscle actin (αSMA) promoter methylation, which is mostly unmethylated in myofibroblast cells (Hu et al., 2010), showed consistent results (Figure S1D). Similarly, flow cytometry analysis of human myometrial and fibroid tissue identified approximately 70%–90% of αSMA-positive smooth muscle cells (Figure S1E). These analyses indicate that our molecular study is a good reflection of smooth muscle cells and not unduly influenced by contaminating cells. Origins of fibroid and normal myometrial samples from specific patients were confirmed by SNP analysis, which identified distinct branches in paired normal myometrium and fibroids whereas isolated branches were restricted to the unpaired samples (Figure S1F).

**Figure 1.**
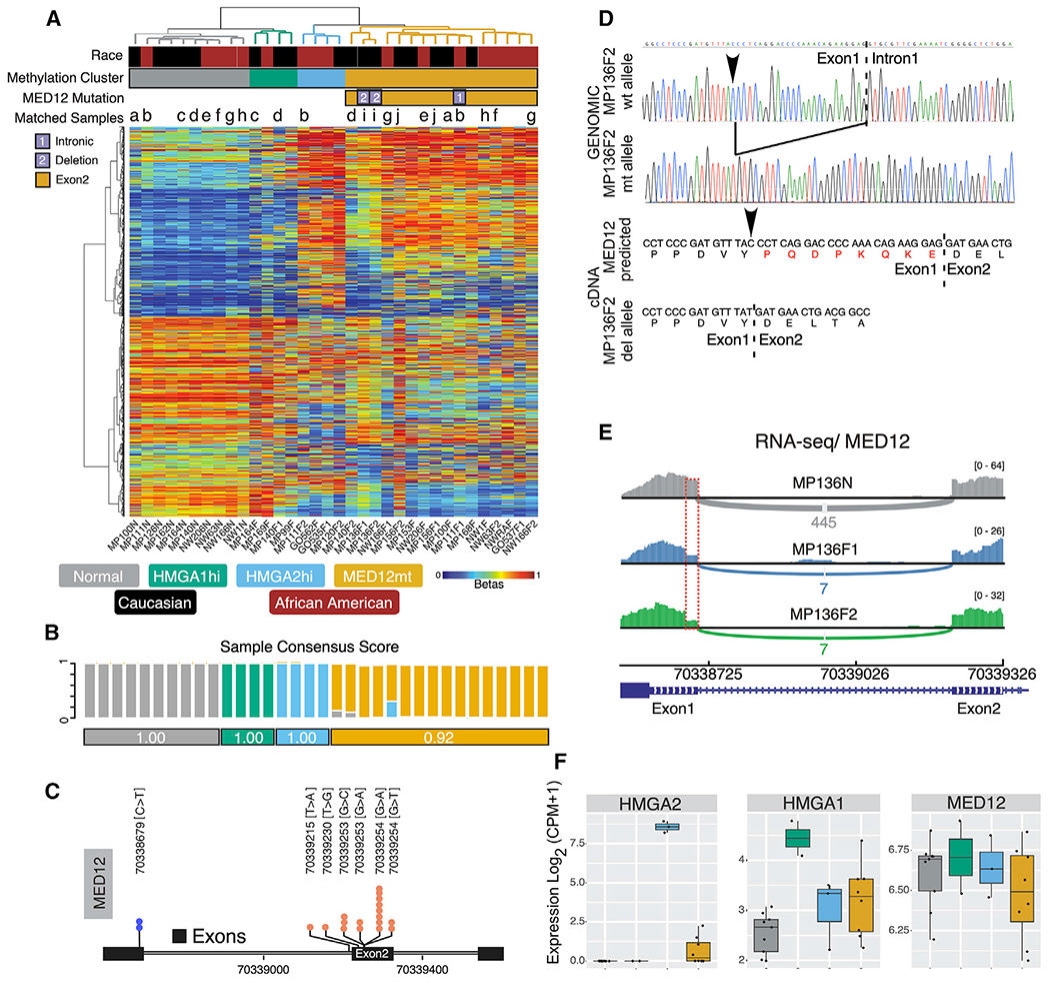
Subtyping of Uterine Fibroids Based on DNA Methylation Profiling (A) Hierarchical clustering of 10 normal and 24 fibroid samples (columns) on 10,000 most variable CpG sites (rows). A color gradient from blue to red in the heatmap indicates a low to high level of methylation (beta values of 0 to 1, corresponding to 0% to100% methylation). Race information for each sample is color-coded in black for Caucasian and red for African American. Samples aggregated into four clusters comprising normal myometria (n = 10) and *MED12*mt (n = 16), *HMGA2*hi (n = 4), and *HMGA1*hi (n = 4) fibroid subtypes. The mutation status of *MED12* is indicated with a column side bar, with yellow indicating canonical exon 2 mutations. Two non-canonical mutationsare indicated by numbers. Multiple samples from the same individual are annotated with the same letter at the top of the heatmap. (B) Sample stability score is shown as stacked bars and defined as the average consensus value between a sample and members of a consensus cluster so that there are multiple scores for a sample at a k = 4 consensus clusters. Stability for each cluster is the average pairwise sample stability score of samples in a consensus cluster and is shown below the sample bar charts. (C) Lollipop plot showing the distribution of different somatic mutations of *MED12*. (D) Sanger sequencing electropherogram confirming the novel 24-bp deletion of *MED12* identified from WES and the C > T mutation (arrowhead). Sequences from two MP136F2 genomic clones are shown. The sequence of mutated cDNA is from the amplified PCR product of MP136F2. (E) RNA-seq read pileup for *MED12* exons 1 and 2, illustrating decreased reads at the 24-bp deletion (dotted red box). (F) Boxplots (boxes, 25%–75%; whiskers, 10%–90%; lines, median) showing mRNA expression for subtype genes for each DNA methylation-based subtype tissue identified in (A).

Unsupervised clustering of DNA methylation data (Figure 1A) of the most variable 1% of CpG sites (Table S1) revealed segregation of normal myometria and fibroids. Fibroids were further split into three major clades in the dendrogram. Although the *HMGA1*hi fibroids clustered with the normal myometrial samples, the *MED12*mt and *HMGA2*hi fibroids clustered closer to each other (Figure 1A). The race of the patient did not appear to influence the clustering results. Consensus clustering analysis (Wilkerson and Hayes, 2010) with 1,000 iterations showed that the three discovered methylation clusters of fibroids were robust, as demonstrated by both sample-based and cluster-based stability scores (Figure 1B). One of the *MED12*mt fibroids showed a relatively high tendency to be clustered with *HMGA2*hi fibroids but still clustered predominantly with other *MED12*mt fibroids.

We analyzed the fibroids for *MED12* mutation status by Sanger sequencing, and hotspot exon 2 mutations in *MED12* (Mäkinen et al., 2011) were detected in most cases (Figure 1C). However, an unreported C > T mutation coupled with a 24-bp deletion from two separate fibroids collected from the same patient (MP136) were also detected using WES and confirmed by Sanger sequencing (Figure 1D). RNA-seq also showed a clear drop in *MED12* reads in the deleted region of these two fibroids compared with the rest of the first exon and to the same region in their matched normal myometrial sample (Figure 1E). Analysis of the cDNA from these fibroids confirmed the C > T mutation and deletion. Splicing of intron 1 did not appear to have been affected, but the observed in-frame deletion did result in mRNA with a predicted translated protein missing 8 amino acids (Figure 1D). *HMGA1* and *HMGA2* overexpression marked the two other groups in the non-*MED12*mt fibroids (Figure 1F). *MED12* expression levels were not significantly different between normal myometria and fibroid subtypes (Figure 1F).

**Figure 2.**
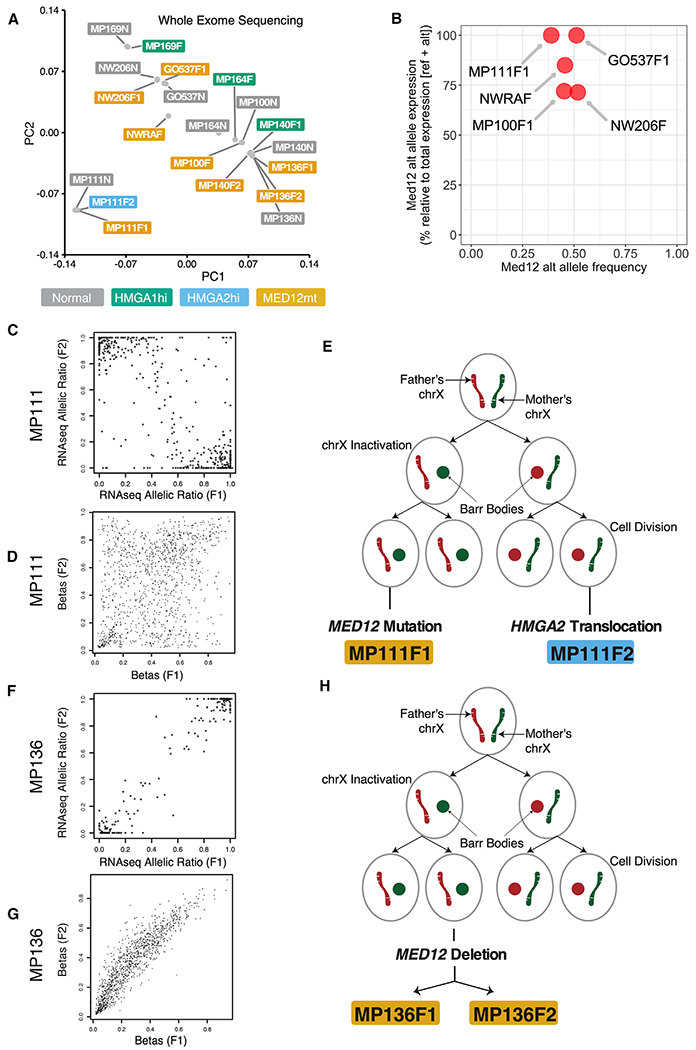
Genomic Landscape Identified by WES of Uterine Fibroids (A) MDS (multidimensional scaling) plot based on WES from *HMGA2*hi (n = 1), *HMGA*1hi (n = 3), and *MED12*mt (n = 8) fibroids and matched normal myometria (n = 8). (B) Dotplot showing the altered allele expression (y axis) and altered allele frequency (x axis) of gene *MED12*. (C and F) Single cell of origin analyses for patient MP111 (C) and MP136 (F) with two fibroids present (F1 and F2). (D and G) Allelic expression for F1 (x axis) and F2 (y axis), based on germline SNPs on chromosome X, was plotted as stars, accompanied by scatterplots showing methylation pattern correlations between F1 (x axis) and F2 (y axis) for patient MP111 (D) and MP136 (G). (E and H) Evolution trees with fibroid subtype events for patients MP111 (E) and MP136 (H).

Multidimensional scaling of WES results showed clustering of the myometria and fibroids by patient, confirming that these were matched samples (Figure 2A). We also confirmed that the mutant *MED12* allele was the expressed allele in the *MED12*mt fibroids analyzed (Figure 2B). With both WES and RNA-seq data, we were able to investigate whether the paired fibroids from the same patients (MP111 and MP136) came from a single cell or had separate origins by examining X chromosome inactivation patterns. One of the two X chromosomes is randomly inactivated early in development, and fibroids with different inactive X chromosomes would be unlikely to come from the same cell of origin. MP111F1 and F2 fibroids expressed alternative alleles for heterozygous loci on chromosome X (Figure 2C) as well as poorly correlated methylation patterns (Figure 2D), suggesting that they originated independently from two cells with a different X chromosome inactivated (Figure 2E). In contrast, MP136F1 and F2 expressed the same alleles (Figure 2F) and displayed highly similar methylation patterns (Figure 2G), suggesting a probable single cell of origin (Figure 2H). Conflicting results regarding clonality have been reported; however, previous WES (Mehine et al., 2014) and these results suggest that clonality can vary among fibroids.

The mutational burden of fibroids was generally less than 0.5/MB (Megabase) (Figure S2A), except for MP164F (2.5/MB), and comparable with that of pediatric leukemias and lymphomas, which represent some of the lowest in human cancers profiled to date (Chalmers et al., 2017). A prevalence for C > A mutation in some of the fibroids (Figure S2B) was observed, different from the common C > T mutation in the CpG context. However, mutation signatures did not reveal any overt differences within and between the *MED12*mt and *HMGA2*hi fibroid subtypes (Figure S2C).

### The DNA Methylation Landscape Is Altered in Fibroids

The overall distribution of CpG and CpH (or non-CpG) methylation was largely unremarkable in all samples (Figure S3A). At loci with fibroid-specific hypermethylation compared with normal myometria, *HMGA1*hi fibroids were closest to normal myometrial samples (Figure S3B), similar to the clustering results with the top 1% of the most variably methylated sites (Figure 1A). *HMGA2*hi and *MED12*mt fibroids had elevated levels of methylation at CpH sites (Figure S3C). *HMGA2*hi fibroids had the highest-level gain of methylation at CpG and CpH sites among all groups, which is consistent with higher expression of the *de novo* DNA methyltransferase, DNMT3A (Figure S3D).

CpG islands were largely unmethylated in both normal myometria and fibroids, and highly methylated domains (HMDs) in myometria remained highly methylated in fibroids (Figure S3E). We also did not observe significant hypomethylation in partially methylated domains (PMDs), even though loss of methylation within PMDs, particularly in the context of WCGW (where W = A or T) without neighboring CpGs (dubbed solo-WCGW), has been suggested to track the accumulation of cell divisions in normal cells and is commonly observed in tissues that have undergone extensive clonal expansion, like cancer (Zhou et al., 2018a). Methylation at enhancer regions, however, exhibited a small shift in the overall distribution between normal myometria and fibroids from PMDs to HMDs (Figure S3E).

Enhancer activity can be inferred from DNA methylation profiles (Yao et al., 2015), with unmethylated distal regions usually marking active enhancers. A large fraction of the probes on the EPIC array interrogate distal elements (defined as ±2 kb away from the transcription start site [TSS]) containing at least one binding site for each of the 158 transcription factors (TFs) we annotated previously (Zhou et al., 2017), based on ENCODE chromatin immunoprecipitation sequencing (ChIP-seq) data (ENCODE Project Consortium, 2012). We assessed enrichment or depletion of differentially methylated cytosines (DMCs) in binding sites for the TFs by hypergeometric testing (false discovery rate [FDR] = 1 × 10^−6^), comparing all fibroids and each of the fibroid subtypes with normal myometria (Figure S4). *HMGA1*hi fibroids were similar to normal myometria and had few DMCs. *MED12*mt and *HMGA2*hi fibroids exhibited similarity in transcription factor binding site (TFBS) enrichment at their hypermethylated distal loci, further indicating that they could share similar transcriptional rewiring. Notably, binding sites for EZH2 and SUZ12, components of polycomb repressive complex 2 (PRC2) (Margueron and Reinberg, 2011), were highly enriched in the hypermethylated, and likely closed-off, cytosines. In contrast, estrogen receptor-α (ERα) binding sites were enriched in distal sites that lose methylation and are presumably activated in fibroids, which is consistent with the steroid hormone dependence of the disease (Bulun, 2013).

### DNA Hypomethylation in the HMGA2 Gene Body of HMGA2hi Fibroids

**Figure 3.**
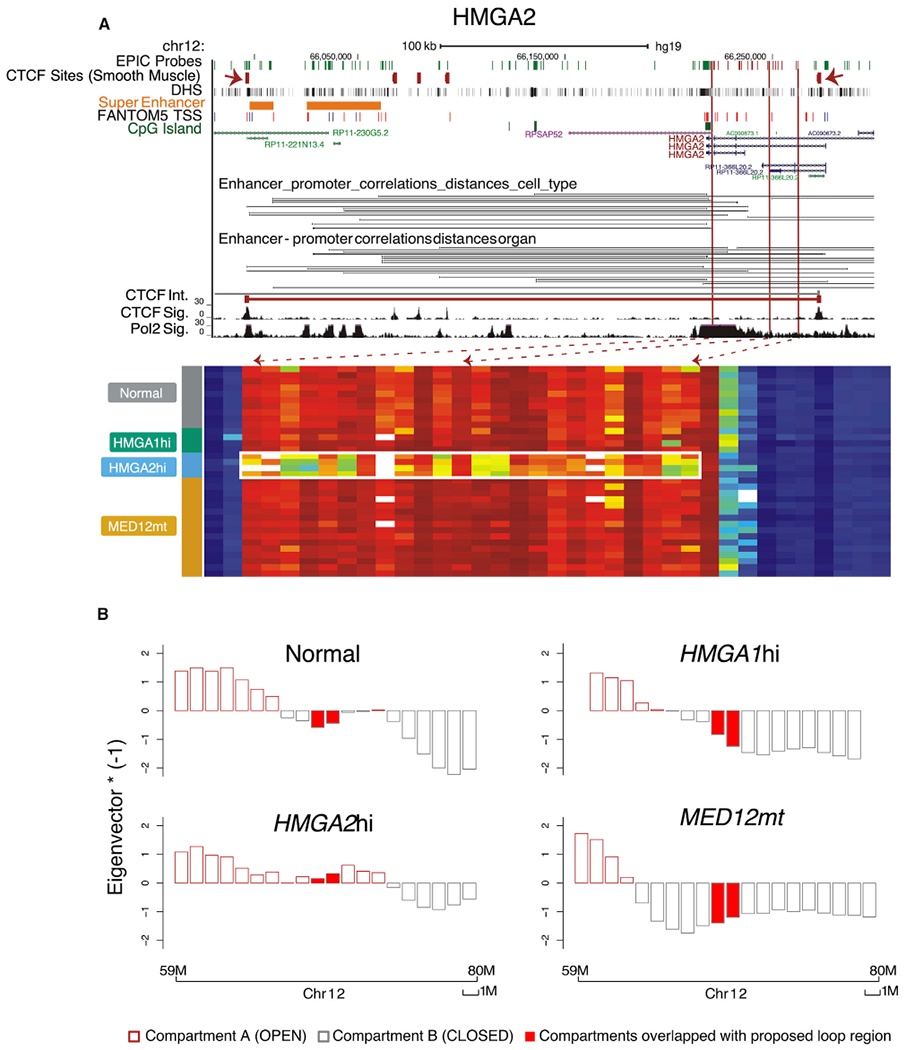
*HMGA2* Gene Body Hypomethylation and Altered Chromatin Organization in *HMGA2*hi Fibroids (A) University of California Santa Cruz (UCSC) genome browser view of gene *HMGA2*, coupled with the DNA methylation level for this region, shown as a heatmap (columns, CpGs; rows, samples grouped by subtype).Top UCSC tracks include locations of EPIC target probes, CTCF ChIP-seq peaks from smooth muscle cells differentiated *in vitro* from H9 embryonic stem cells, DNase I hypersensitivity sites (DHSs), predicted super enhancer sites, and CpG island, UCSC gene, and FANTOM5-curated TSS, and enhancer-promoter correlations. The CTCF signal and RNA polymerase II (Pol II) signal in the MCF-7 cell line are added on top of the gene heatmap together with CTCF ChIA-PET interactions from the ENCODE project. The heatmap shows the differentially methylated region in the *HMGA2* gene in *MED12*mt (n = 16), *HMGA2*hi (n = 4), and *HMGA1*hi (n = 4) fibroids, and normal myometria (n = 10). The white box in the heatmap highlights the location of gene body hypomethylation in fibroids with the *HMGA2*hi subtype. Red dashed arrows indicate the corresponding genomic locations of those hypomethylated probes. Two red solid arrows in the CTCF ChIP-seq peak track show the location of CTCF in smooth muscle cells, in line with the predicted CTCF ChIA-PET interaction boundaries. (B) Reconstruction of A/B compartments using EPIC array data on chromosome 12 in each of the four DNA methylation groups. The eigenvector is plotted with directions flipped by multiplying with negative 1 to show open compartments in the positive orientation. The predicted CTCF loop region marked in (A) is indicated with filled red bars, which switches from the B to the A compartment specifically in the *HMGA2*hi subtype.

Because *HMGA1* and *HMGA2* overexpression is observed in approximately 20%–30% of all fibroids, we chose to further analyze DNA methylation in these genes. Compared with normal myometria, two adjacent CpG sites in a CpG island within the *HMGA1* promoter gained DNA methylation in *MED12*mt and *HMGA2*hi fibroids but remained hypomethylated in *HMGA1*hi fibroids (Figure S5A; Figure 1F). In contrast, a segment of the gene body of *HMGA2* (measured by 13 consecutive DNA methylation probes) was hypomethylated in *HMGA2*hi fibroids compared with other fibroids and normal myometria (Figure 3A). This hypomethylated region was also observed in a uterine fibroid cell line with an *HMGA2* translocation (Carney et al., 2002) and the one *HMGA2*hi fibroid in a validation set of fibroid samples (Figures S5B and S5C). Upregulation of *HMGA2* expression has been generally attributed to rearrangements at 12q14-15 (Ashar et al., 1995; Schoenmakers et al., 1995). However, when fluorescence *in situ* hybridization (FISH) was performed on fresh-frozen tumor sections or exponentially growing fibroid cell cultures of samples identified as *HMGA2*hi, 12q14-15 rearrangement was not detected in either GO535F1 or MP120F2 with probes spanning over 600 kb upstream of *HMGA2* (Figures S6A and S6B). qRT-PCR was performed to confirm assignment of these fibroids to the *HMGA2*hi subtype by the RNA-seq results (Figure S6C). The 3′ end distal hypomethylated CpG site was located near a binding site for CCCTC-binding factor (CTCF) as determined by ENCODE ChIP-seq data (Figure 3A; Wang et al., 2012b). CTCF is involved in forming long-range chromatin loops that alter the 3D structure of chromosomes and acts as an insulator of transcriptional activity of the encompassed genes (Wang et al., 2012a). To locate CTCF binding regions upstream of and within the *HMGA2* gene body, we analyzed enhancer-promoter interactions from FANTOM5 (Figure 3A, black lines) and overlaid it with available ChIA-PET interaction data (Figure 3A, red line). We also inferred open (A) and closed (B) compartments (A/B compartments) (Fortin and Hansen, 2015) using DNA methylation profiles surrounding the *HMGA2* region (Figure 3B). Indeed, the chromatin was open for this locus in the *HMGA2*hi subtype specifically and closed in the other subtypes and in normal myometria. These results suggest that hypomethylation could be an additional mechanism allowing overexpression of *HMGA2*, at least in some fibroids.

### Transcriptome Analyses Identifies Commonalities and Differences between Fibroid Subtypes

**Figure 4.**
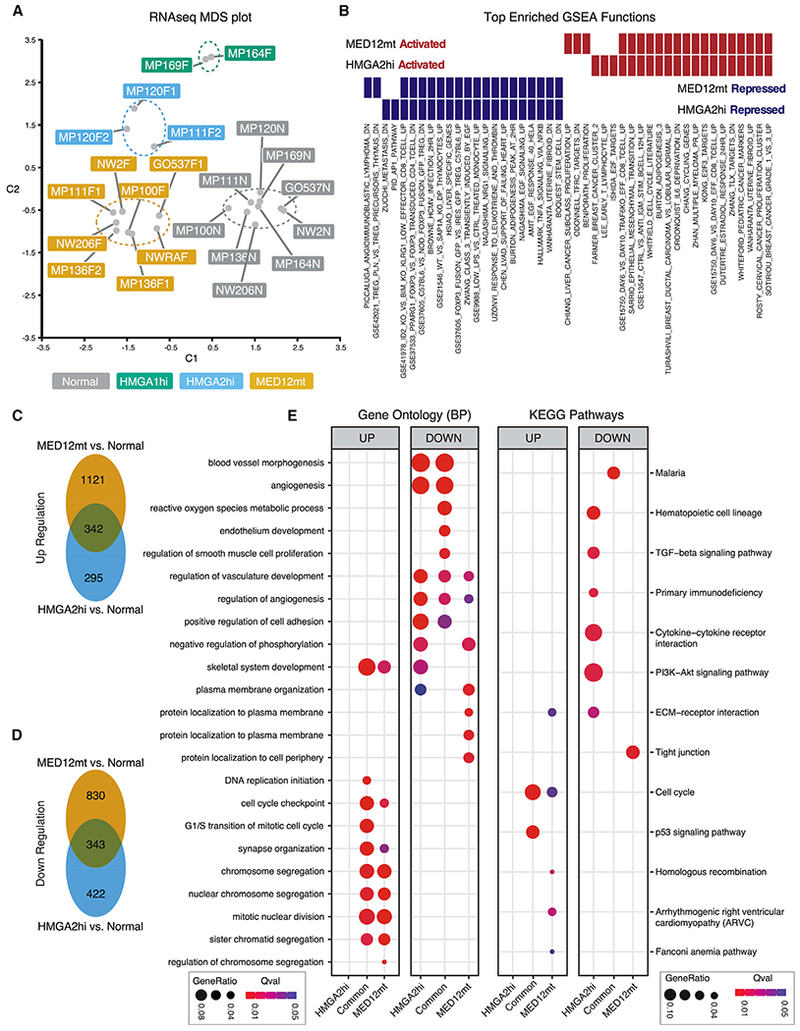
Transcriptome Characterization of Fibroid Subtypes (A) MDS plot based on RNA-seq data, showing the first two dimensions. Each dot represents one sample, colored by DNA methylation-based subtypes: *MED12*mt (n = 8), *HMGA2*hi (n = 3), and *HMGA1*hi (n = 2) fibroids and normal myometria (n = 9). (B) GSEA analysis of up- or downregulated genes in *MED12*mt and *HMGA2*hi subtypes compared with normal samples. The top 20 enriched gene sets for genes upregulated (red) or downregulated (blue) in each fibroid subtype versus normal myometrium are shown. (C and D) Venn diagrams illustrate the overlap for upregulated (C) and downregulated genes (D) between *MED12*mt and *HMGA2*hi subtypes. (E) Gene Ontology and KEGG pathways analyses for *HMGA2*hi-specific, shared, and *MED12*mt-specific up- and downregulated gene sets. Gene enrichment ratio and significance level are shown by the size and color of each circle, respectively.

As with DNA methylation, global RNA-seq analyses showed that the *MED12*mt, *HMGA1*hi, and *HMGA2*hi subtypes also clustered separately from each other and from normal myometria independent of patient origin (Figure 4A). Gene set enrichment analyses (GSEAs) of the RNA-seq results between the *MED12*mt or *HMGA2*hi fibroids, when each was compared with normal myometria, showed a large number of shared activated and repressed genes among the top-ranked gene sets (Figure 4B). More than half of the upregulated genes in *HMGA2*hi fibroids were similarly regulated in the *MED12*mt fibroids (Figure 4C), and nearly half of the downregulated genes in *HMGA2*hi fibroids were also downregulated in *MED12*mt fibroids (Figure 4D). Gene Ontology analyses of the differentially expressed genes (DEGs) showed a high concordance of dysregulated genes (Figure 4E). Kyoto Encyclopedia of Genes and Genomes (KEGG) pathway analyses of the DEGs showed that a few of the pathway changes were shared between *MED12*mt and *HMGA2*hi fibroid subtypes. As reported in previous transcriptomics profiling of fibroids, we also identified elevated expression of *RAD51B, PLAG1*, and *PAPPA2* in our *MED12*mt, *HMGA2*hi, and *HMGA1*hi fibroids, respectively (Table S2; Mehine et al., 2016). These RNA-seq results suggest that the *MED12*mt and *HMGA2*hi fibroid subtypes are more alike transcriptomically than they are different.

In Figure 1F, we showed that the average *HMGA2* expression level was also elevated in *MED12*mt fibroids (Log2 fold change [FC] = 3.2) but to a lesser degree than in *HMGA2*hi fibroids (Log2 FC = 11.6). More granular examination of the RNA-seq results showed that, although some of the *MED12*mt fibroids did not express *HMGA2*, other *MED12*mt fibroids did show elevated levels of *HMGA2* expression (Figure S7A), suggesting that there might be further heterogeneity within *MED12*mt fibroids. However, these *HMGA2*-expressing, *MED12*mt fibroids did not cluster closer to the *HMGA2*hi fibroids in either the RNA-seq (Figure 4A) or DNA methylation heatmaps (Figure 1A). Thus, the physiological significance of activation of *HMGA2* expression in some *MED12*mt fibroids is not clear.

**Figure 5.**
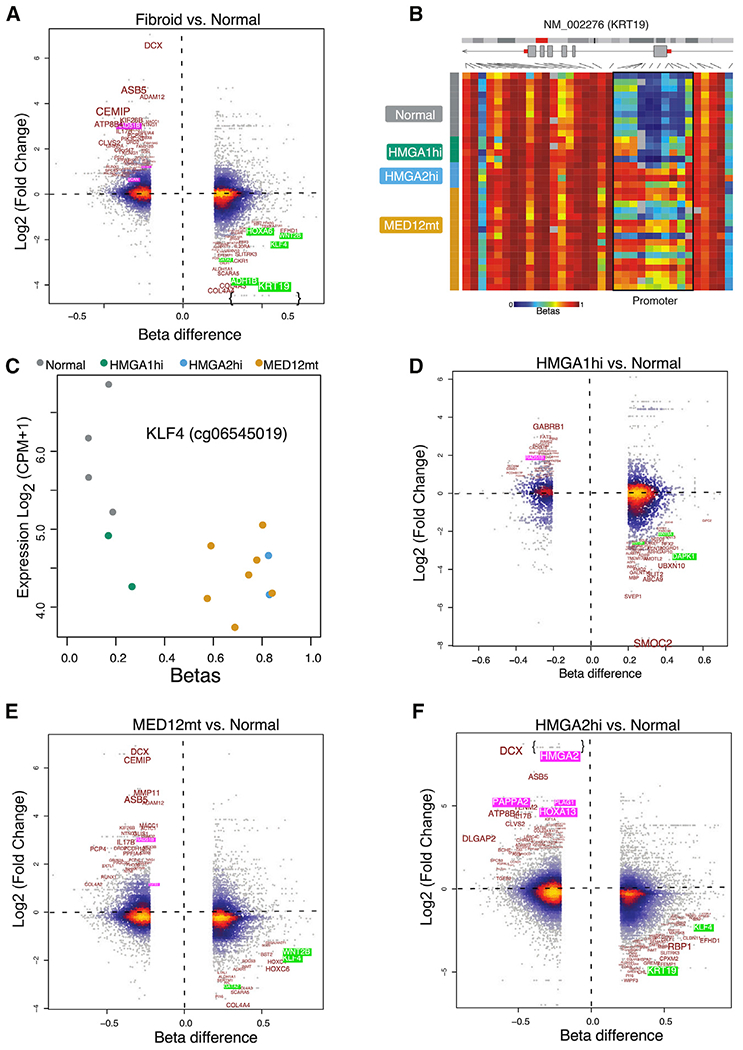
Differential Promoter DNA Methylation versus Differential Gene Expression (A and D–F) Promoter DNA methylation beta difference is plotted on the x axis, and Log2 fold change for the corresponding gene is plotted on the y axis for MED12mt (n = 7), HMGA2hi (n = 2), HMGA1hi (n = 2), and normal myometrial (n = 4) samples with both methylation and expression results. This analysis is done for all fibroids (A) and for *HMGA1*hi (D), *HMGA2*hi (E), and *MED12*mt (F) versus normal samples. Color represents local dot density. Green-highlighted genes are some of the most highly correlated between promoter hypermethylation and reduced expression. Pink-highlighted genes are some of the most highly correlated between promoter hypomethylation and induced expression. The text is sized to account for both probe methylation difference and corresponding gene fold change. (B) *KRT19*, an example of a gene likely silenced by DNA methylation, is further visualized as a heatmap of *MED12*mt (n = 16), *HMGA2*hi (n = 4), and *HMGA1*hi (n = 4) fibroids and normal myometria (n = 10), with multiple probes called within the same gene. (C) Dotplot (probe methylation is plotted as the x axis and related gene expression as the y axis) of KLF4, showing that downregulation of gene expression is observed in all fibroid subtypes compared with normal myometria.

To identify genes dysregulated because of altered promoter methylation, we integrated DNA methylation and RNA-seq profiles for each fibroid subtype (Figure 5). Genes with significantly altered promoter CpG methylation (absolute delta β > 0.25, p < 0.05) and an associated gene expression change between normal myometrium and fibroids are listed in Table S3. A few hypomethylated and upregulated genes were identified, but most (*VCAN, RAD51B, COL1A1*, etc.) have been reported previously (Behera et al., 2007; Mehine et al., 2013; Norian et al., 2009) and are not described further. *KRT19*, which has also been reported previously to be silenced by promoter hypermethylation in fibroids (Navarro et al., 2012), was the most downregulated gene in all fibroids compared with normal myometria using this analysis (Figure 5A). The heatmap of β values for the EPIC probes in the *KRT19* gene show that most of the hypermethylation in fibroids is occurring in the promoter and exon 1 regions (Figure 5B). We also identified many additional genes silenced similarly by promoter methylation, including genes involved in the retinoic acid pathway (*ADH1B*), WNT pathway (*WNT2B*), and stem cell function (*GATA2* and *KLF4*) (Figure 5A). Many of these are known tumor suppressor genes whose silencing could be important for fibroid growth. Analysis of *KLF4* showed that it was hypermethylated and downregulated in each of the fibroid subtypes compared with normal myometria (Figure 5C). We then analyzed coordinated differential methylation and gene expression by fibroid subtypes. In *HMGA1*hi fibroids, *SMOC2* was the most hypermethylated and downregulated gene (Figure 5D). SMOC2 can stimulate endothelial cell proliferation and migration, which is consistent with the hypoxia observed in fibroids (Casey et al., 2000; Mayer et al., 2008). *MED12*mt fibroids (Figure 5E) had a differential gene pattern similar to that of total fibroids. Several of the DEGs (*PAPPA2* and *PLAG1*, for example) in *HMGA2*hi fibroids have also been described previously. However, *HOXA13* was identified to be hypomethylated and upregulated in *HMGA2*hi fibroids compared with normal myometria (Figure 5F). Given the importance of this homeobox gene in female reproductive tract development, we continued to investigate the HOXA locus further.

### HOXA13 Overexpression Correlates with Homeotic Transformation in Myometrium

**Figure 6.**
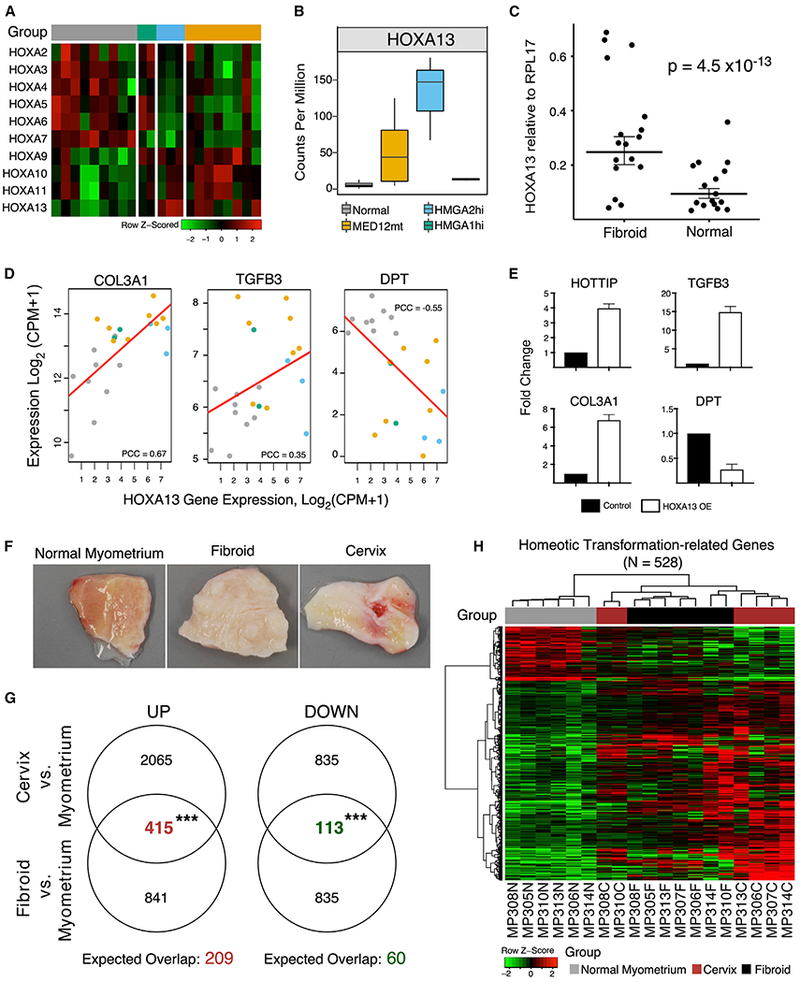
*HOXA13* Overexpression in Normal Myometrium Suggests Homeotic Transformation (A) RNA-seq results in the HOXA cluster show a switch from *HOXA2-7* to *HOXA9-13* expression in normal myometria (n = 9) compared with *MED12*mt (n = 8), *HMGA2*hi (n = 3), and *HMGA1*hi (n = 2) fibroids. (B) Boxplots (boxes, 25%–75%; whiskers, 10%–90%; lines, median) showing *HOXA13* gene expression from the RNA-seq results in normal myometria and *MED12*mt, *HMGA2*hi, and *HMGA1*hi fibroids in (A). (C) Relative expression of *HOXA13* byqRT-PCR compared with the RPL17 housekeeping gene in another set of samples between normal myometria (n = 17) and fibroids (n = 19), analyzed for statistical significance by mixed-effect regression with random intercepts (p = 4.52 × 10^−13^). Top and bottom lines, 25%–75%; middle line, median. (D) RNA-seq for *HOXA13* (x axis) versus that of fibroid-characteristic genes (y axis of each panel for *COL3A1, TGFB3,* and *DPT*) in each of the samples described in (A). Red lines represent linear regression trends, and the Pearson’s correlation coefficient (PCC) is indicated for each plot. (E) qRT-PCR analyses of *HOTTIP, TGFB3, COL3A1*, and *DPT* mRNA expression in control untransfected UT-TERT cells compared with UT-TERT cells transfected with HOXA13. Mean fold change normalized to control is shown, with error bars representing SEM of three independent experiments. (F) The similarity in appearance of typical uterine fibroids and cervical tissues compared with normal myometrium is shown by gross analyses. (G) Venn diagrams showing the overlap of DEGs between fibroids and normal myometrium and between normal cervical stroma and normal myometrium. DEGs are split into up- and downregulated genes, with numbers labeled in the diagrams. The numbers of both sets of overlapping genes, as colored in red and green, are significantly higher than expected by chance, indicated below each diagram, by chi-square tests (***p < 0.001). (H) Overlapping up- and downregulated DEGs (528 total) are shown in the heatmap, with rows representing genes and columns representing matched samples of unsubtyped fibroids (n = 7), normal myometria (n = 6), and normal cervical stromata (n = 7). Sample group information is color-coded and shown on top of the heatmap. Gene expression is scaled on rows and capped at ± 2.

HOXA genes encode a supercluster of homeobox TFs that are highly conserved and critical regulators of proper development of the female reproductive tract (Daftary and Taylor, 2006). Along the cranial-to-caudal axis of the differentiating Müllerian duct, *HOXA10* is expressed in the uterus, whereas *HOXA13* is expressed in the cervix and vagina (Kobayashi and Behringer, 2003; Taylor et al., 1997), and replacement of the *HOXA11* gene with *HOXA13* in the mouse uterus leads to homeotic transformation to a more posterior phenotype (Zhao and Potter, 2001). This homeotic transformation confirms that *HOXA11* and *HOXA13* are not functionally redundant while strengthening the role of HOXA genes in female reproductive tract differentiation. Based on RNA-seq data, the fibroids in this study switched to expression of more posterior HOXA genes compared with normal myometria (Figure 6A). Among the HOXA genes, only *HOXA13* mRNA expression reached statistical significance after correcting for multiple comparisons in a genome-wide survey. It was highly expressed in *MED12*mt (Log2 FC = 3) and *HMGA2*hi (Log2 FC = 4.4) fibroids compared with either normal myometria or *HMGA1*hi fibroids (Figure 6B). We confirmed high *HOXA13* mRNA abundance in another, unsubtyped set of fibroid samples compared with adjacent normal myometria by qRT-PCR (Figure 6C). The long non-coding RNA (lncRNA) HOXA-transcript at the distal end (*HOTTIP*), which is located at the 5′ end of the HOXA cluster and is coordinately regulated with that of distal HOXA genes (Wang et al., 2011), was also elevated in fibroids (Figures S7B and S7C). qRT-PCR revealed a high correlation between *HOXA13* and *HOTTIP* mRNA levels in normal myometrium and uterine fibroids (Figure S7D), suggesting coordinated expression or even a potential interrelated feedforward mechanism driving uterine fibroid differentiation.

The expression of a number of smooth muscle cell and extracellular matrix genes is known to be altered in uterine fibroids compared with myometria. *HOXA13* expression in fibroids positively correlates with the expression levels of *COL3A1* (Malik et al., 2010) and *TGFB3* (Malik et al., 2010) and negatively correlates with that of *DPT* (Arslan et al., 2005, genes associated with the fibroid phenotype (Figure 6D). *HOXA13* overexpression in the UT-TERT myometrial cell line led to significantly altered expression of these genes and that of *HOTTIP* compared with control untransfected UT-TERT cells (Figure 6E), indicating that HOXA13 can likely regulate the expression of these characteristic fibroid genes.

**Figure 7.**
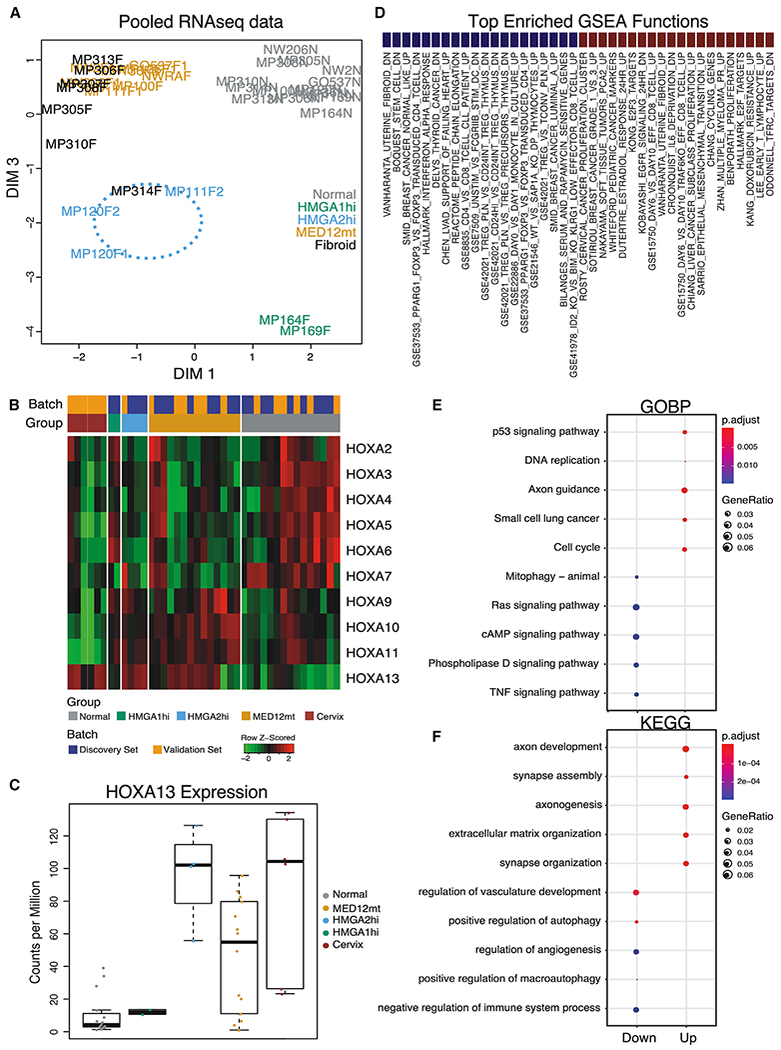
Transcriptome Characterization of the Pooled Discovery and Validation Set (A) MDS plot based on RNA-seq gene expression data. Each label represents one sample of *MED12*mt (n = 14), *HMGA2*hi (n = 4), and *HMGA1*hi (n = 2) fibroid subtypes and normal myometria (n = 15). Black labels indicate fibroids from the validation set. (B) Gene expression patterns for the HOXA gene family in samples from (A) along with normal cervical stromata (n = 6). Batch information is colored by discovery or validation set. Group information is colored by methylated-based subtypes and cervical stroma category. (C) Boxplots (boxes, 25%–75%; whiskers, 10%–90%; lines, median) of *HOXA13* gene expression in fibroid subtypes and cervical stroma samples in (B). (D) Top 20 significantly enriched gene sets in GSEA for genes up-regulated (right, blue) and down-regulated (left, red) in fibroids compared to matched normal myometria in the validation set. (E and F) Gene Ontology Biological Process (GOBP, Panel E) and KEGG pathway (KEGG, Panel F) enrichment analysis for genes likely to be implicated in the homeotic transformation process. Upregulated genes (right; 415 in total) and downregulated genes (left; 113 in total) are as determined in Figure 6G. Gene enrichment ratio and significance level are shown by the size and color of the corresponding circle, respectively.

Both uterine fibroids and cervical stroma are characterized by a significant amount of extracellular matrix (Islam et al., 2013; Leppert et al., 2014; Winkler and Rath, 1999), and we observed that uterine fibroids appear grossly similar to normal cervical stroma (Figure 6F), which is consistent with a hypothetical homeotic change in fibroids to a more cervical phenotype. To assess whether a potential homeotic transformation occurs in fibroids, normal myometria, normal cervical stroma, and fibroids from the same individuals (n = 7) were collected and profiled by RNA-seq (Figure 7A). The HOXA gene expression levels of the samples matched the previous set of samples (Figure 7B), including *HOXA13* (Figure 7C), and appeared somewhere between normal myometria and cervical stroma. We identified DEGs between fibroids and normal myometrium and between normal cervical stroma and normal myometrium (Figure 6G). By splitting DEGs into up- and downregulated DEGs, we observed a more significant overlap between DEGs in both directions than expected by chance. These overlapping DEGs (N = 528; Table S4) are likely associated with the phenotypic similarity of fibroids and cervical stroma and with possible homeotic transformation. Indeed, visual examination with a heatmap showed that uterine fibroids exhibit strong similarity with normal cervical stroma and form one joint cluster in this subspace of the transcriptome, separate from normal myometrium (Figure 6H). When we analyzed the biological processes associated with the DEGs, developmental pathways were most commonly observed, suggesting that differentiation or dedifferentiation mechanisms were activated (Figures 7D and 7E). Together, these results support the notion that development of uterine fibroids is, in fact, at least a partial homeotic transformation into a more cervical phenotype, probably by induced expression of *HOXA13* in normal myometrial cells.

## DISCUSSION

A genetically modified mouse model has been developed that shows that expression of the *MED12* mutant transgene on either a *MED12*-null or WT background leads to fibroid formation, suggesting that *MED12* mutation could drive their development through a gain-of-function (GOF) or dominant-negative mechanism (Mittal et al., 2015). In contrast, biochemical assays demonstrate that *MED12* mutations lead to loss of CycC-CDK8/19 binding and function, suggesting that *MED12* mutations might be a loss-of-function (LOF) phenotype (Park et al., 2018). Biochemical analysis further revealed quantitative differences within the various *MED12* exon 2 mutations with regard to kinase activity, indicating that maybe all *MED12* mutants are not equal (Park et al., 2018). In our study, we discovered mutations distinct from the more common mutations in exons 1 and 2 (Mäkinen et al., 2011), a 24-bp deletion at the end of exon 1, and a 44-bp deletion spanning the wild-type splice acceptor in exon 2, resulting in deletion of 8 and 15 amino acids, respectively (Figure 1; Figure S7E). These deletions are both in-frame, which, together with the canonical hotspot mutations, suggests against a LOF model. Interestingly, fibroids with the non-canonical *MED12* mutations clustered with other *MED12* mutants in both DNA methylation and RNA-seq analyses (Figures 1 and 4), suggesting that these mutations might be functionally equivalent to the more common *MED12* mutations.

One of the limitations of this study is that WES was used to determine the mutational burden. WES was used by The Cancer Genome Atlas (TCGA) and other studies, and a wide range of mutational burdens has been observed across various samples examined (Lawrence et al., 2013). Despite its broad use, concerns regarding non-uniform distribution of genome coverage, and genotyping quality still present considerable challenges for downstream bioinformatics analyses. Similarly, mutation signature analysis with WES is not as precise and comprehensive as whole-genome sequencing (WGS) (Alexandrov et al., 2013). In addition, with the overall low mutation load, technical noise in next generation sequencing (NGS) can overshadow a specific mutation signature, if any, operative in these fibroids. A much higher proportion of false-positive variants has also been reported in WES (Belkadi et al., 2015; Fuentes Fajardo et al., 2012), which can further bias downstream mutation load determination. Others have performed WGS for bulk tissue and for specific populations (reviewed in Commandeur et al., 2015; Moravek and Bulun, 2015) to mitigate these concerns.

Overexpression of *HMGA2*, a gene normally not highly expressed in normal myometrium, is the second most common phenomenon known to occur in fibroids (Sandberg, 2005). *HMGA2* overexpression has been attributed to genetic alterations involving translocations or aberrant splicing within the coding region (Quade et al., 2003; Schoenberg Fejzo et al., 1996). *HMGA2* expression also appears to be regulated by the microRNA Let-7 family (Peng et al., 2008; Wang et al., 2007). A GOF mechanism involving fusion of *RAD51B* to *HMGA2* has also been correlated with overexpression (Takahashi et al., 2001); however, we were unable to detect *RAD51B-HMGA2* fusion or aberrant splicing in our WES and RNA-seq analyses. Using FISH probes spanning increasing distances upstream of the *HMGA2* gene body, translocations were confirmed in only one of the *HMGA2*hi fibroids analyzed. The gene body hypomethylation we observed in all *HMGA2*hi fibroids could be another mechanism involved in *HMGA2* upregulation. The hypomethylation might be associated with altered CTCF-mediated looping, which would facilitate interaction between a distal enhancer and the *HMGA2* gene promoter, leading to overexpression of *HMGA2*. Biochemical confirmation of CTCF binding and activation of *HMGA2* gene expression in uterine fibroids will need to be performed to confirm this mechanism of *HMGA2* upregulation. Alternatively, this hypomethylation could be the result of an unidentified upstream event such as translocation, either as an intermediate step to or caused by *HMGA2* overexpression.

Enhancers have been described as the most dynamically used component of the genome (Calo and Wysocka, 2013; Shlyueva et al., 2014; Sur and Taipale, 2016). Previously published genome-scale DNA methylation studies utilized the Illumina Infinium HumanMethylation27 (Navarro et al., 2012) or HumanMethylation450 (Maekawa et al., 2013) arrays, which largely focused on the promoter regions. The EPIC array boasts unparalleled coverage of enhancer sites (Zhou et al., 2017). Binding sites for the PRC2 proteins EZH2 and SUZ12 were most enriched for hypermethylated sites in fibroids compared with normal myometria. PRC2 is involved in reversible transcription repression and has been characterized most extensively (Simon and Kingston, 2013) as a master regulator of stem cell differentiation. PRC2 binding sites have been shown to be highly enriched for sites hypermethylated in cancer (Widschwendter et al., 2007) and presumably maintain a “locked-in” stem-like signature in malignant cells. Although recent studies (Yang et al., 2016) and our study (Table S2) have shown higher expression of *EZH2* in uterine fibroids, analyses of altered *EZH2* expression and binding and the resulting changes in target gene expression in uterine fibroids to determine mechanisms of action and possible therapeutic potential have yet to be reported. Our analysis also identified the binding sites for NANOG, a TF pivotal for self-renewal and ground state pluripotency of embryonic stem cells (Boyer et al., 2005), were also selectively methylated in both *MED12*mt and *HMGA2*hi fibroids. *KLF4*, one of the Yamanaka pluripotency-inducing factors, could also silenced by DNA methylation in fibroids (Figure 5C), which is consistent with a diminished role of stem cells in fibroids (Chang et al., 2010; Ono et al., 2012). We and others have hypothesized that fibroids evolve from myometrial stem cells that have undergone genetic modifications that drive differentiation away from their stem cell state (Bulun, 2013; Commandeur et al., 2015). The loss of KLF4 expression and closed NANOG binding sites observed in this study both support this hypothesis. We also identified hypomethylated DNA in TFBSs of ERα (*ESR1*) in all fibroid subtypes, except for *HMGA1*hi, and of the glucocorticoid receptor (GR; *NR3C1*) (Figure S4). A number of studies have reported higher expression of *ESR1* in uterine fibroids compared with normal myometria (Benassayag et al., 1999; Kovács et al., 2001; Otsuka et al., 1989), and treatment of cultured fibroid cells with estrogen can increase proliferation and cell cycle progression (Barbarisi et al., 2001). In contrast, GR, another member of the nuclear steroid receptor superfamily, has been postulated to be antagonistic to the estrogen-induced response in fibroids (Bever et al., 1956; Bitman and Cecil, 1967; Rhen et al., 2003). *HMGA2*hi fibroids have been reported to be larger in size compared with *MED12*mt tumors (Heinonen et al., 2017; Hennig et al., 1999). Perhaps hypomethylation in GR TFBSs in *MED12*mt fibroids antagonizes estrogen-induced effects, such as controlling fibroid size. ChIP-seq and functional assays will need to be performed to determine whether altered methylation at these TFBSs affects fibroid biology in a meaningful way.

Our results strongly suggest that expression of *HOXA13* in uterine fibroids drives aberrant gene expression in myometrial cells, transforming them into uterine fibroids with a gene expression profile more similar to cervical stroma. Much like fibroids, the cervix, a collagen-dense tissue that protects the uterus from pathogenic assault, undergoes significant remodeling postpartum (Islam et al., 2013; Leppert et al., 2014; Winkler and Rath, 1999). Thus, development and resolution of fibroids could be controlled by mechanisms similar to those observed in the cervix. For example, progesterone can keep the cervix stiff and help prevent cervical softening or ripening (Larsen and Hwang, 2011) and is normally required for fibroid growth and development (Ishikawa et al., 2010). At term, the mechanisms driving cervical ripening, characterized by partial dissolution of the collagen matrix, which is necessary for delivery (Timmons et al., 2010), could also be driving the decrease in fibroid burden observed in postpartum uteri (Baird and Dunson, 2003). During parturition, the lower uterine segment of the myometrium undergoes a regionalization event leading to increased contractility, a fundamental aspect of spontaneous labor (Challis et al., 2000). This contractile phenotype has now been attributed to higher expression of *HOXA13*, a key regulator of a number of genes that are involved in cell contractility and cell-cell adhesion, further associating a role of *HOXA13* with myometrial transformation (Liu et al., 2015).

In conclusion, we characterized the genetic and DNA methylation profiles of uterine fibroids, which allowed their subtyping. We also showed high correlation with gene expression and DNA methylation, highlighting the regulatory potential of altered DNA methylation driving uterine fibroid development. TFBS analysis identified fibroid subtype-specific regulators and hints at a critical role of these regulators in fibroid tumorigenesis. Finally, integrated characterization of our DNA methylation and RNA-seq results showed a switch in HOXA gene expression in fibroids, suggesting a homeotic transformation of normal myometrium to cervical stroma-like tissue in fibroid etiology, probably through subtype-independent upregulation of *HOXA13* expression.

## STAR★METHODS

### LEAD CONTACT AND MATERIALS AVAILABILITY

Further information and requests for resources and reagents should be directed to and will be fulfilled by the Lead Contact, Jose Teixeira (teixei15@msu.edu). The HOXA13 overexpression plasmid generated in this study has been deposited with Addgene (LV-hHOXA13).

### EXPERIMENTAL MODELS AND SUBJECT DETAILS

#### Cell Lines

The human myometrial (UT-TERT) and fibroid (GM-TERT) cell lines were a kind gift from Dr. John Risinger and have been characterized previously (Carney et al., 2002). UT-TERT cells were cultured and maintained in SmGM™-2 Smooth Muscle Growth Medium-2 containing 5% FBS, 0.1% insulin, 0.2% basic human fibroblast growth factor (hFGF-b), 0.1% GA-100, and 0.1% human epidermal growth factor (hEGF) (Lonza, Walkersville, MD).

#### Human Samples

Human tissues were collected from macrodissection of hysterectomies from consented, reproductive age women using Spectrum Health Systems or Northwestern University IRB approved protocols for secondary use of biobank. Human patient sample characteristics can be found in Table S5.

### METHOD DETAILS

#### Sample Processing

Fibroids (n = 26) and matched normal myometria (n = 15) samples in the discovery set are described in Table S5A. Tissues with the same numbered identifier were from the same patient. If the last letter was N, this was the normal myometrium or F, this was fibroid tissue. If the F was followed by a number, this indicates more than one fibroid from the same patient was analyzed. A validation set includes matched normal myometrium, fibroid, and cervical stroma from the same individual (n = 7 each for RNA-seq of each tissue type; n = 9 each for EPIC array for each tissue type) were collected through the same process as the discovery set (Table S5B). Sample names in the validation set ending with a C indicate cervical stroma. One myometrial sample, MP307N, and one cervical sample, MP305C, were removed from analyses due to poor sample qualities, according to the pathology reports. Fibroids samples were collected from fibroids < 6 cm in diameter to avoid excess extracellular matrix and at random locations in the uterus to attempt capture of all clinical possibilities. All analyses were done with one or two random fibroids from each patient to avoid over-representation. Samples were aliquoted upon arrival for DNA and RNA isolation, and cell separation. Myometrial samples were confirmed to be histologically normal. African American and Caucasian samples were confirmed by SNP probes on the EPIC array with R package, SeSAMe (Zhou et al., 2017). *MED12*mt fibroids were defined by mutation in either exons1 or 2. *HMGA1*hi and *HMGA2*hi expression were defined by not having a *MED12* mutation in exons 1 or 2 and expression > 2-fold and > 5-fold, respectively, by qRT-PCR compared to matched normal. *MED12* mutation was determined by PCR amplification followed by Sanger sequencing using primers 5′-CTTCGGGATCTTGAGCTACG-3′ and 5′-GGAGGGTTCCGTGTAGAACA-3′ for Exon1, primers 5′-GCTGGGAATCCTAGTGACCA-3′ and 5′-GGCAAACTCAGCCACTTAGG-3′ targeting Exon 2. *MED12* cDNA was amplified using primers 5′-CTTCGGGATCTTGAGCTACG-3′ and 5′-AAGCTGACGTTCTTGGCACT-3′ spanning Exon 1 and Exon 2. *HMGA1* AND *HMGA2* overexpression was determined by RNA-seq and/or confirmed by qRT-PCR with the following primers 5′-GAAGTGCCAACACCTAAGAGACC-3′ and 5′-GGTTTCCTTCCTGGAGTTGTGG-3′ and 5′-GAAGCCACTGGAGAAAAACGGC-3′ and 5-GGCAGACTCTTGTGAGGATGTC-3′, respectively. MP308F *MED12* deletion was confirmed by Sanger sequencing. Promoter (defined as ± 500bp of transcription start site (TSS)) methylation status of miR-200 family (Park et al., 2008; Vrba et al., 2010) and alpha smooth muscle actin (α-SMA) (Skalli et al., 1989) were also employed to validate the major component of the cells analyzed as smooth muscle.

#### Flow Cytometry

Tissues (two matched normal and fibroid samples) were minced, placed in digestion media (DMEM/F12, 1X antibiotic-antimycotic, 10% FBS, 2 mg/ml Collagenase Type I (Sigma), 1 mg/ml DNase Type I (Sigma), and 5 mM MgCl2 and incubated at 37°C overnight with agitation. Cell suspensions were passed through 100 μm and 40 μm cell strainers, washed with PBS, and centrifuged. The cell pellets were resuspended in 5 mL ACK Lysing Buffer (Thermo Fisher Scientific) to remove red blood cells, washed with PBS, centrifuged, and resuspended in 2% PFA for 10 min. Following PBS wash and centrifugation, cells were permeabilized in ice-cold methanol. Cells were washed again then incubated with anti-α-smooth muscle-cy3 (Sigma, cat #C6198,1:500) in PBS with 0.5% NP-40 for 1.5 h at RT. Cells were washed and resuspended in PBS and analyzed on an Accuri C6 flow cytometer (BD Biosciences, San Jose, CA). Events were gated initially by forward and side scatter, then for singlets (side scatter area × height) and finally for Cyanine 3 (Cy3) fluorescence using FlowJo software (FlowJo, Ashland, OR). Unstained cells served as a gating control.

#### Fluorescence *In Situ* Hybridization (FISH)

FISH probes were prepared from purified BAC clones from the BACPAC Resource Center (bacpac.chori.org). The BAC clones are as follows; Probe set 1: CH17-111D2 (12q14.3-1 green) and CH17-63I9 (12q14.3-2 orange), Probe set 2: CH17-392C11 (12q14.3-3 green) and CH17-63I9 (12q14.3-2 orange), and Probe set 3: CH17-305B19 (12q14.3-5 green) and CH17-63I9 (12q14.3-2 orange). FISH probe 12q14.3-2 is located at the distal end of gene *HMGA2*, while probes 12q14.3-1, 12q14.3-3, and 12q14.3-5 are all proximal of *HMGA2*. FISH probe 12q14.3-2 was labeled with Orange-dUTP and all other probes were labeled with Green-dUTP (Abbott Molecular Inc., Abbott Park, IL), by nick translation. Tumor touch preparations were prepared by imprinting thawed tumors onto positively-charged glass slides. The sample slides were fixed in methanol:acetic acid (3:1) for 30 min and air-dried. Slides were then aged in 2X saline/sodium citrate (SSC) at 60°C for 20 min, digested with 0.005% pepsin at 37°C for 5 min, and washed with 1X PBS for 5 min. Slides were placed in 1% formaldehyde/PBS for 10 min at room temperature, washed with 1X PBS for 5 min, and dehydrated in an ethanol series (70%, 85%, 95%) for 2 min each. Slides were then denatured in 70% formamide/2X SSC at 74°C for 3.5 min, washed in a cold ethanol series (70%, 85%, 95%) for 2 min each, and air-dried. The FISH probes were denatured at 75°C for 5 min and held at 37°C for 10-30 min until 10 ul of probe was applied to each sample slide. Slides were coverslipped and hybridized overnight at 37°C in the ThermoBrite hybridization system (Abbott Molecular Inc.). The posthybridization wash was with 2X SSC at 73°C for 3 min followed by a brief water rinse. Slides were air-dried and then counterstained with VECTASHIELD mounting medium with 4’-6-diamidino-2-phenylindole (DAPI) (Vector Laboratories Inc., Burlingame, CA). Image acquisition was performed at 1000x system magnification with a COOL-1300 SpectraCube camera (Applied Spectral Imaging-ASI, Vista, CA) mounted on an Olympus BX43 microscope. Images were analyzed using FISHView v7 software (ASI) and 20 interphase nuclei were scored for each sample.

#### Whole-Exome Characterization of Uterine Leiomyomas and Matched Normal Tissues

Genomic DNA from all uterine fibroids and corresponding normal myometrium were extracted from freshly frozen tissue using DNeasy Blood and Tissue kit (QIAGEN) according to manufactures recommendation. Samples were submitted to Hudson Alpha (Huntsville AL) for 2 × 100 sequencing on an Illumina HiSeq 2500. In total, 8 fibroids and matched normal tissue pairs were sequenced. Three out of the eight sample pairs had an additional fibroid sample sequenced. Exome capture was performed using the NimbleGen SeqCap EZ Exome v3 kit and sequenced to a depth of approximately 45x across two flowcells. Reads were assessed for quality using FastQC v0.11.5 and MultiQC v1.0dev0. Samples were called for germline and somatic variants using the Broad Institute’s “Best Practices” guidelines with GATK v3.6. Briefly, reads were aligned to the human genome (hg19) using BWA mem with the -M and -R options to mark short, split alignments as secondary and add read group information, respectively. Next, SAM files were converted to coordinate sorted BAM files using samtools v1.3.1, keeping the header (-h) and aligned reads (-F 4). Picard Tools v2.7.1 was used to mark and remove duplicates with the MarkDuplicates functionality and REMOVE_DUPLICATES = true. For germline variant calling, known variant files included: dbSNP build 149, 1000 genomes phase 3, and Mills and 1000 genomes gold standard indels. Interval reference files (design files) for the NimbleGen SeqCap EZ Exome v3 kit were downloaded from the NimbleGen website 04 November, 2016. Specifically, the SeqCap_EZ_Exome_v3_hg19_primary_targets.bed was used with the interval padding option set to 100 bp unless specified otherwise. De-duplicated, aligned reads were subjected to base quality score recalibration with the-filter_mismatching_base_and_quals and BadCigar filters in place. Calling germline variants was accomplished using HaplotypeCaller with the BadCigar filter in place and emitting a Genomic VCF (GVCF). GVCFs for each sample were combined and genotyped using the GATK GenotypeGVCFs functionality. SNPs were filtered using a QD < 2.0, FS > 60.0, MQ < 40.0, Mapping-QualityRankSum < −12.5, ReadPosRankSum < −8.0. Indels were filtered using a QD < 2.0, FS > 200.0, ReadPosRankSum < −20.0.

For somatic variant calling, a panel of normals was generated using Mutect2 for each normal sample in artifact detection mode, passing dbSNP, COSMIC (v71), and the NimbleGen SeqCap EZ Exome v3 interval list (SeqCap_EZ_Exome_v3_hg19_primary_targets.bed) with the interval padding option set to 100 bp. For the panel of normals, variants were kept if it was observed in at least two normal samples and not filtered (e.g.–filteredAreUncalled–filteredrecordsmergetype KEEP_IF_ANY_UNFILTERED). Variants were called using Mutect2 for each fibroid and normal sample pair passing dbSNP, COSMIC, panel of normals, and the NimbleGen SeqCap EZ Exome v3 interval list to GATK.

Variant annotation was performed on germline and somatic variants using vt (v0.5), VEP (release 87), vcfanno (v0.1.0), and gemini (v0.19.1) in conjunction with COSMIC, ClinVar (Downloaded 12 December 2016), and ExAC databases (Release 0.3.1). First, multiallelic sites were decomposed and variants normalized using vt. Next, variants were annotated using VEP (Ensembl) and then by vcfanno. A pedigree file (.ped) was generated using the following line: grep -m1 CHROM input.v*cf*. | cut -f 10- | awk ‘BEGIN{RS = “\t”}{ printf(“fam%d\t%s\t0\t0\t-9\t-9\n,” NR, $1)}’ > output.ped. The pedigree file and annotated VCF were then used to generate the gemini database with the vcf2 db.py (https://github.com/quinlan-lab/vcf2db). Finally, the annotated variants were filtered and queried against the COSMIC, ClinVar, and ExAC databases using gemini. VCFs were converted to maf using VEP and vcf2maf (https://github.com/mskcc/vcf2maf). Somatic variation was visualized using R (v3.3.2) and maftools (v1.0.55). Mutational signatures inferred from single nucleotide variants were done using R package SomaticSignatures (Gehring et al., 2015). WES raw data are made available through SRA (Sequence Read Archive) under SRA identifier SRP163897.

#### Illumina Infinium HumanMethylation BeadChip Assay Characterization

Genome-scale DNA methylation was profiled by the HumanMethylationEPIC (EPIC) BeadChip (Illumina, CA, USA), which interrogates a total of 863,904 CpG loci spreading across the transcription start sites and enhancer/regulatory regions. Additionally, 2932 non-CpG loci (CpH) and 59 single nucleotide polymorphisms (SNPs) are also included as part of the EPIC array. In detail, DNA was quantified by Qubit fluorimetry (Life Technologies) and 500ng of DNA from each sample was bisulfite-converted using the Zymo EZ DNA Methylation Kit (Zymo Research, Irvine, CA USA) following the manufacturer’s protocol using the specified modifications for the Illumina Infinium Methylation Assay. After conversion, all bisulfite reactions were cleaned using the Zymo-Spin binding columns, and eluted in Tris buffer. Following elution, BS converted DNA was processed through the EPIC array protocol. To perform the assay, converted DNA was denatured with NaOH, amplified, and hybridized to the EPIC bead chip. An extension reaction was performed using fluorophore-labeled nucleotides per the manufacturer’s protocol. Array beadchips were scanned on the Illumina iScan platform.

Raw IDAT files were processed using R package SeSAMe (Zhou et al., 2018b) with noob background correction (Triche et al., 2013), non-linear dye bias correction, and non-detection masking. DNA methylation beta values were called as quantitative percentage of methylated signals over both unmethylated and methylated signals. Beta values range from 0 to 1, with “0” indicating complete lack of methylation and “1” full methylation. We excluded measurements from sub-optimally designed probes due to overlap with SNPs and repeat elements, as suggested by previous studies (Zhou et al., 2017). Raw IDAT files are available through GEO (Gene Expression Omnibus) database under accession GSE120854 and GSE135446.

#### DNA Methylation Analysis

Unsupervised hierarchical clustering was conducted based on most variable probes (top 1% standard deviations out of all the CpG probes) across all samples measured on the EPIC array, and was visualized as heatmap with continuous betas. In order to evaluate the robustness of sample membership in the discovered methylation clusters, we performed consensus clustering by perturbing samples for 1000 iterations (Wilkerson and Hayes, 2010). Sample- and cluster-based stability score were then calculated using R package ConsensusClusterPlus (Version 1.48.0; (Wilkerson and Hayes, 2010)). The sample- and cluster-based stability score provide a way to quantitatively present the probability of samples being assigned to a certain cluster, and that of a discovered cluster. Fibroids-specific methylation profiling was generated using CpG probes unmethylated in normal myometria but methylated (defined by a beta value of no less than 0.3) in at least one sample within each fibroid subtype. Hierarchical clustering based on betas within each subtype was then performed to show the specific methylation patterns across different subtypes. Methylation-based compartment A/B calling was conducted using R package minfi at resolution of 100kb (Fortin and Hansen, 2015). Overall dimension deduction plot was generated using UMAP (Uniform Manifold Approximation and Projection for Dimension Reduction) (Becht et al., 2018).

Differentially methylated cytosines (DMCs) were called using R package DMRcate (Peters et al., 2015) by comparing all the fibroids, and each fibroid subtype to myometria, based on its build-in default p value cutoff of 0.05 and an absolute beta-value difference threshold at 0.2. DMCs were mapped to genes captured by RNA-seq, if they were located within gene promoters (defined as 2 kb flanking regions surrounding transcription start sites). We then performed enrichment analysis of transcription factors binding sites (TFBSs) at distal regulatory elements (i.e., enhancers). Distal probes were identified as probes located within TFBSs but not within gene promoters.

#### Evaluating Clonality with X Inactivation

For patients with more than one fibroid sample examined, we evaluated the possibility that they arose as independent clones or from the same origin. We performed this analysis by integrating WES and RNA-seq data. For each patient, we identified germline SNPs on the X chromosome from WES data using GATK. We restricted the analyses to those SNPs that remained heterozygous in DNA in both tumors. We then examined which alleles (A or B) were expressed for these SNPs using RNA-seq data. Alternative expressed alleles would indicate separate cellular origins, as random inactivation of one X chromosome occurs early in development.

#### Transcriptomic Profiling of Uterine Fibroids and Matched Normal Tissues

Total RNA was extracted using Trizol Reagent (Invitrogen) according to manufactures instructions, from freshly frozen samples stored at −80C. The RNA was suspended in RNase-free water, and purified with an RNeasy MinEluteTM clean up kit (QIAGEN). RNA concentration and integrity were assessed using a Nanodrop 1000 spectrophotometer (Thermo Scientific, Wilmington, Delaware) and Agilent 2100 Bioanalyzer (Agilent Technologies, Santa Clara, California), according to manufacturer’s protocol. Seventeen samples were submitted to the Van Andel Research Institute (VARI) Genomics Core for 2 × 75 bp RNA-seq on an Illumina NextSeq 500. Libraries were prepared using a Kapa RNA HyperPrep Kit with ribosomal reduction, pooled, and sequenced across two flowcells to yield approximately 50-60 million reads/sample. Reads were assessed for quality using FastQC v0.11.5 and MulitQC v1.0dev0. Next, raw reads for the sample were merged from two flowcells into a single file and aligned to the human genome (hg19) with STAR v2.5.2b using the two-pass mode. Transcript abundance was quantified using HTSeq v0.6.1p1 with the–stranded option set to “reverse” and Ensembl GTF (Release 75) as the annotation file. Validation set was processed following the same procedure to make the gene expression comparable. Differential expression (DE) was calculated using either edgeR (v3.16.5) for comparing fibroids to myometria; or limma (v3.30.13) for comparing *HMGA2*hi, and *MED12*mt fibroids to myometria. Counts were filtered to include genes with a minimum of 1 count per million (CPM) in at least 3 samples. Differentially expressed genes were identified as those having an FDR less than 0.05 relative to the comparator. MDS plots were generated in R (v3.3.2) using R package ggplot2 (v2.2.1). Expressed somatic variants identified from exomes were determined using the Broad Institute’s “Best Practices” for RNA-seq variant calling. Briefly, we added read group information (using function AddOrReplaceReadGroup from Picard Tools) to BAM files generated by STAR with two-pass mode, and then sorted them by coordinates. Picard Tools v2.7.1 was used to mark duplicates using function MarkDuplicates. Known variant files passed to the exomes were used as known sites in RNA-seq variant calling procedure with GATK v3.6. Cigar strings were modified using function SplitNCigarReads in GATK with the ReassignOneMappingQuality function (RMQF 255, RMQT 60, and -U ALLOW_N_CIGAR_READS). Interval targets were generated and indels realigned with GATK. De-duplicated and indel realigned reads were then subjected to base quality score recalibration. After recalibration, these BAMs were fed to HaplotypeCaller to call variants with filters dontUseSoftClippedBases enabled and stand_call_conf set at 20.0. SNPs and indels were further filtered by -window 35, -cluster 3, FS > 30.0, and QD < 2.0. Expressed somatic variants were identified using bedtools (v2.26.0) with both annotated RNA-seq variants and exome variants. SRA accession numbers for fastq files are SRP166862 and SRP217468.

#### Publicly Accessible Datasets and Bioinformatic Tools

Gene set enrichment analysis was conducted using gene sets downloaded from The Molecular Signatures Database (MSigDB) (Subramanian et al., 2005) excluding collection of computational gene sets (C4) and gene ontology gene sets (C5). Top 20 enriched GSEA terms were shown. TFBS-probe annotation of Illumina EPIC array (human reference genome (NCBI build 37/Hg19)) was download from Zhou et al. (2017). Particular gene view was generated using UCSC genome browser with tracks available from track hubs (Kent et al., 2002), including EPIC probe coordinates, CpG islands, super enhancer annotations (Wei et al., 2016), FANTOM5-curated TSS locations and enhancer-promoter correlations, DNase peaks, and ENCODE-curated Pol2 and CTCF binding sites, together with CTCF ChIA-PET interactions in cell line MCF7, and CTCF ChIP-seq binding sites from female embryo (5 days) smooth muscle *in vitro* differentiated cells originated from H9 (ENCODE Mar 2012 Freeze). The Integrative Genomics Viewer (IGV) was employed to examine and view aligned sequence reads (Robinson et al., 2011). Functional analysis, for example Gene Ontology (GO) and Kyoto Encyclopedia of Genes and Genomes (KEGG) analysis were carried out using R package clusterProfiler (PMID: 22455463), with an FDR cutoff of 0.05.

#### Cell Culture and Nucleofection

*HOXA13* overexpression plasmid, pLV(Exp)-EGFP:T2A:Puro-CBh > hHOXA13, vector ID VB180306-1076naw, was constructed and packaged by VectorBuilder (Cyagen Bioscience). Nucleofection was carried out using Amaxa Basic Nucleofector Kit for Primary Mammalian smooth muscle cells (Lonza, Catalog # VPI-1004) according to manufactures protocol. Briefly, 1X10^6^ cells was resuspended in 100μl Nucleofector solution, and transfected with 1μg of *HOXA13* overexpression plasmid using program P-024. Following nucleofection, cells were incubated for 18 hours and media was changed to complete growth media along with supplements. 48 hours after nucleofection, the cells were treated with 2μg/ml puromycin to select for stable expression. Expression analysis was determined by qRT-PCR on cloned cells (n=4) with *RPL17* as the housekeeping gene.

#### Quantitative Real Time PCR

Total RNA was isolated and treated with Dnase from UT-TERT and HOXA13-UT-TERT clones using an RNA extraction kit (QIAGEN, Valencia, CA). cDNA was synthetized from 1 μg of total RNA using SuperScript IV Reverse Transcriptase (Invitrogen). Quantitative Real time PCR (qRT-PCR) analysis using SYBRGreen (BioRad) was performed to analyze gene expression using the ViiA 7 qPCR System (Applied Biosystems). RPL17 was used for normalization. Primer sequences (5′-3′) used for qRT-PCR are *HOXA13* FP (TGGAACGGCCAAATGTACTGCC), *HOXA13* RP (GGTATAAGGCACGCGCTTCTTTC), *DPT* FP (GCCCATATTCCTGCTGGCTAA), DPT RP (GTGGTTGTTGCTCCTCGGAT), *COL3A1* FP (TGGTCTGCAAGGAATGCCTGGA), COL3A1 RP (TCTTTCCCTGGGACAC CATCAG), *TGFB3* FP (CTAAGCGGAATGAGCAGAGGATC), *TGFB3* RP (TCTCAACAGCCACTCACGCACA), *HOTTIP* FP (CCTAAAGCCACGCTTCTTTG), *HOTTIP* RP(TGCAGGCTGGAGATCCTACT), *RPL17* FP (ACGAAAAGCCACGAAGTATCTG), *RPL17* RP (GACCTTGTGTCCAGCCCCAT). The fold change in gene expression was calculated using the standard ΔΔCt method.

### QUANTIFICATION AND STATISTICAL ANALYSIS

For hyper- or hypo-DMC probe set generated from each comparison between fibroid subtypes and normal samples, hypergeometric test was applied to calculate the enrichment or depletion of binding sites for each TF within DMC set at distal probes. Significance cutoff was made at 1e-6 after false discovery (FDR) correction. Differential expression (DE) was calculated using either edgeR (v3.16.5) for comparing fibroids to myometria; or limma (v3.30.13) for comparing *HMGA2*hi, and *MED12*mt fibroids to myometria. Counts were filtered to include genes with a minimum of 1 count per million (CPM) in at least 3 samples. Differentially expressed genes were identified as those having a FDR < 0.05 relative to the comparator. Average gene expression was measured in triplicate by quantitative real time PCR of fibroid and normal tissue samples and calculated by the ΔΔCt method. *HOXA13* and *HOTTIP* expression were then normalized to the housekeeping gene *RPL17*. Kendall’s tau was used to determine the amount of concordance between mean *HOXA13* and *HOTTIP* expression in both fibroid and normal tissues. To determine if mean gene expression normalized to *RPL17* differed between normal and fibroid tissues, a linear mixed-effects model with a random intercept for each patient was fit. All the analyses were performed using R software with versions newer than 3.4.1 (https://cran.r-project.org/) (R Development Core Team, 2006).

### DATA AND CODE AVAILABILITY

The datasets generated in this study are available at NCBI without restriction. The accession numbers for the RNA sequencing data reported in this paper are [SRA]: [SRP166862; SRP217468]. https://www.ncbi.nlm.nih.gov/sra/?term=SRP166862; https://www.ncbi.nlm.nih.gov/sra/?term=SRP217468; The accession number for the whole exome sequencing data reported in this paper is [SRA]: [SRP163897]. https://www.ncbi.nlm.nih.gov/sra/?term=SRP163897; The accession numbers for the methylation array data reported in this paper are [GEO]: [GSE120854; GSE135446].https://www.ncbi.nlm.nih.gov/gds/?term=GSE120854; https://www.ncbi.nlm.nih.gov/gds/?term=GSE135446.

## Supplementary Material

Figures S1-S7

Table S1

Table S2

Table S3

Table S4

Table S5

Table S6

## Figures and Tables

**Table:** KEY RESOURCES TABLE

REAGENT or RESOURCE	SOURCE	IDENTIFIER
Antibodies		
anti-α-smooth muscle-cy3; clone 1A4	Sigma	Cat# C6198; RRID: AB_476856
Biological Samples		
Hysterectomy samples	Spectrum Health Universal Biorepository, Grand Rapids, MI and Gynecological Biorepository, Department of Obstetrics and Gynecology, Feinberg School of Medicine Northwestern University, Chicago IL	N/A
Critical Commercial Assays		
Methylation EPIC array	Illumina	WG-317-1003
NimbleGen SeqCap EZ Human Exome v3	Roche	6465692001
Kapa RNA HyperPrep Kit	Roche	KK8505
Deposited Data		
RNA-seq Fastq data	This paper	https://www.ncbi.nlm.nih.gov/sra/?term=SRP166862; https://www.ncbi.nlm.nih.gov/sra/?term=SRP217468
Exome-seq Fastq data	This paper	https://www.ncbi.nlm.nih.gov/sra/?term=SRP163897
Methylation raw IDAT	This paper	https://www.ncbi.nlm.nih.gov/gds/?term=GSE120854; https://www.ncbi.nlm.nih.gov/gds/?term=GSE135446
Human reference genome H19, GRCh37	Genome Reference Consortium	https://www.ncbi.nlm.nih.gov/assembly/GCF_000001405.13/
Experimental Models: Cell Lines		
GM-TERT, UT-TERT	John Risinger, PhD, Michigan State University	Carney et al., 2002
Oligonucleotides		
PCR Primers:	This paper	See Table S6.
Recombinant DNA		
BAC clones: CH17-111D2, CH17-392C11, Ch17-305B19, CH17-6319	https://www.bacpac.chori.org/	N/A
Plasmid: pLV(Exp)-EGFP:T2A:Puro-CBh > hHOXA13	This paper	N/A
Software and Algorithms		
SeSAMe	Zhou et al., 2018b	https://www.ncbi.nlm.nih.gov/pubmed/30085201
FastQC V0.11.5	Brown et al., 2017	https://github.com/pnnl/fqc
MultiQC v1.0dev0	Ewels et al., 2016	https://multiqc.info
GATK v3.6	McKenna et al., 2010	https://software.broadinstitute.org/gatk/
BWA mem algorithm	Li and Durbin, 2010	https://github.com/lh3/bwa
Samtools v1.3.1	Li et al., 2009	http://www.htslib.org
Picard v2.7.1	“Picard Toolkit.” 2019. Broad Institute, GitHub Repository. Broad Institute	https://broadinstitute.github.io/picard/
vcf2maf	Mayakonda et al., 2018	https://github.com/mskcc/vcf2maf
Maftools v1.0.55	Mayakonda et al., 2018	https://bioconductor.statistik.tu-dortmund.de/packages/3.5/bioc/vignettes/maftools/inst/doc/maftools.html
R studio v3.3.2, v3.4.1 and v3.4.4	R Development Core Team, 2006	https://www.r-project.org
STAR v2.5.2b	Dobin et al., 2013	https://github.com/alexdobin/STAR/releases
HTSeq v0.6.1p1	Anders et al., 2015	https://www-huber.embl.de/HTSeq
Bedtools v2.26.0	Quinlan and Hall, 2010	http://code.google.com/p/bedtools
The Integrative Genomics Viewer (IGV)	Robinson et al., 2011	http://software.broadinstitute.org/software/igv/
Other		
Molecular Signatures Database (MSigDB)	Subramanian et al., 2005	http://software.broadinstitute.org/gsea/msigdb/index.jsp
UCSC genome browser	Kent et al., 2002	http://genome.ucsc.edu
EPIC array probe annotations	Zhou et al., 2017	https://zwdzwd.github.io/InfiniumAnnotation

## Highlights

- Epigenetic and gene expression profiles define molecular subtypes of uterine fibroids
- DNA hypomethylation in the HMGA2 gene body is consisten with activation of the gene
- HOXA gene cluster expression displays a more posterior pattern in uterine fibroids
- Homeotic transformation appears to play a role in fibroid biology
