# Supplementary material for: Integrated Epigenome, Exome, and Transcriptome Analyses Reveal Molecular Subtypes and Homeotic Transformation in Uterine Fibroids
Source: Cell Rep. Author manuscript; Available in PMC 2020 Jan 13. (PMC6956710; doi:10.1016/j.celrep.2019.11.077)
Supplement: Figures S1-S7 [file NIHMS1547123-supplement-Figures_S1-S7.pdf]

Cell Reports, Volume *29*

Supplemental Information

Integrated Epigenome, Exome, and Transcriptome Analyses Reveal Molecular Subtypes and Homeotic Transformation in Uterine Fibroids

Jitu Wilson George, Huihui Fan, Benjamin Johnson, Tyler James Carpenter, Kelly Katherine Foy, Anindita Chatterjee, Amanda Lynn Patterson, Julie Koeman, Marie Adams, Zachary Brian Madaj, David Chesla, Erica Elizabeth Marsh, Timothy Junius Triche, Hui Shen, and Jose Manuel Teixeira

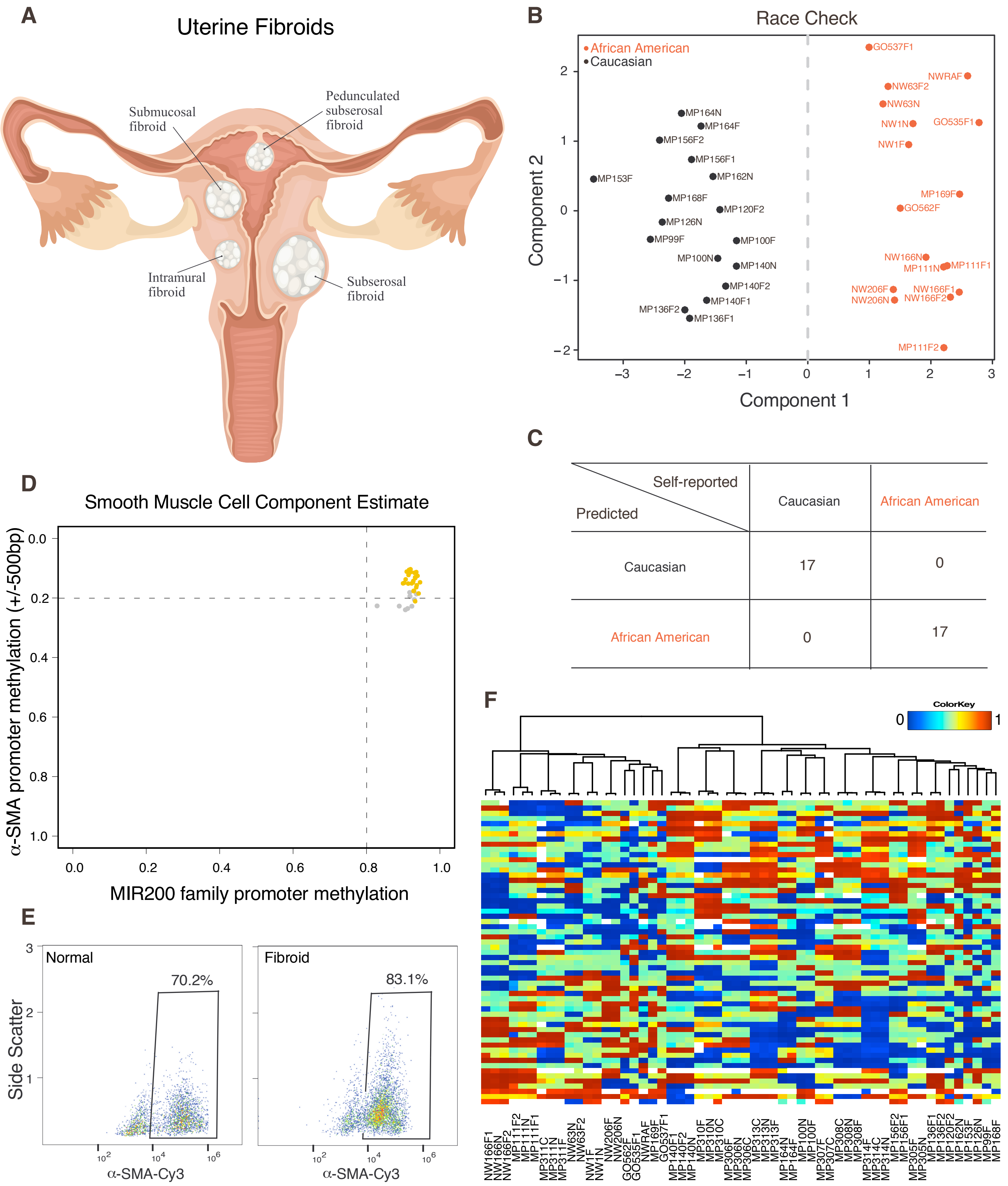

Figure S1. Sample sanity check, Related to STAR Methods. (A) Schematic diagram of the female reproductive tract indicating locations of uterine fibroids. **(B)** Principal component analysis using DNA methylation SNP probes separates African American (n=17) from Caucasian samples (n=17) as predicted by R package, SeSAMe (Zhou et al., 2017). **(C)** Concordance between self-reported and predicted ethnicity in A. **(D)** Percentage of smooth muscle component predicted from promoter methylation of αSMA (y axis) and MIR200 family (x axis). **(E)** Typical FACS sorting demonstrates smooth muscle percentage in both normal and fibroids samples with antibodies to αSMA. **(F)** Heatmap of EPIC SNP probes confirms origin of patient samples (n=56).

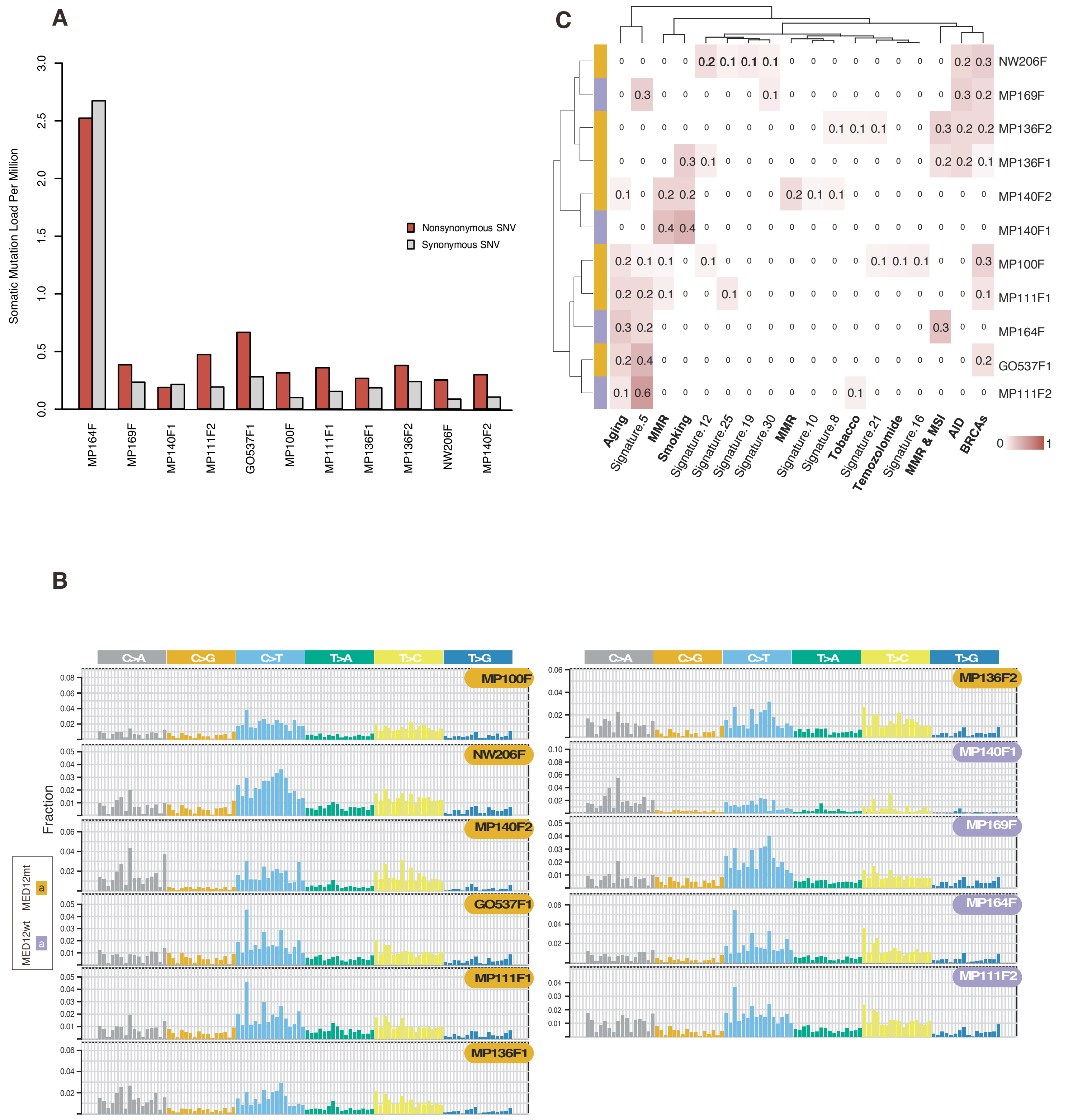

Figure S2. **Mutational landscape of uterine fibroids, Related to Figure 2.** **(A)** Barplot showing mutation load for each fibroid, with synonymous mutations in grey and nonsynonymous in red. **(B)** COMSIC mutation signatures per fibroid. **(C)** Heatmap of signature contributions, with signatures as columns and fibroids as rows.

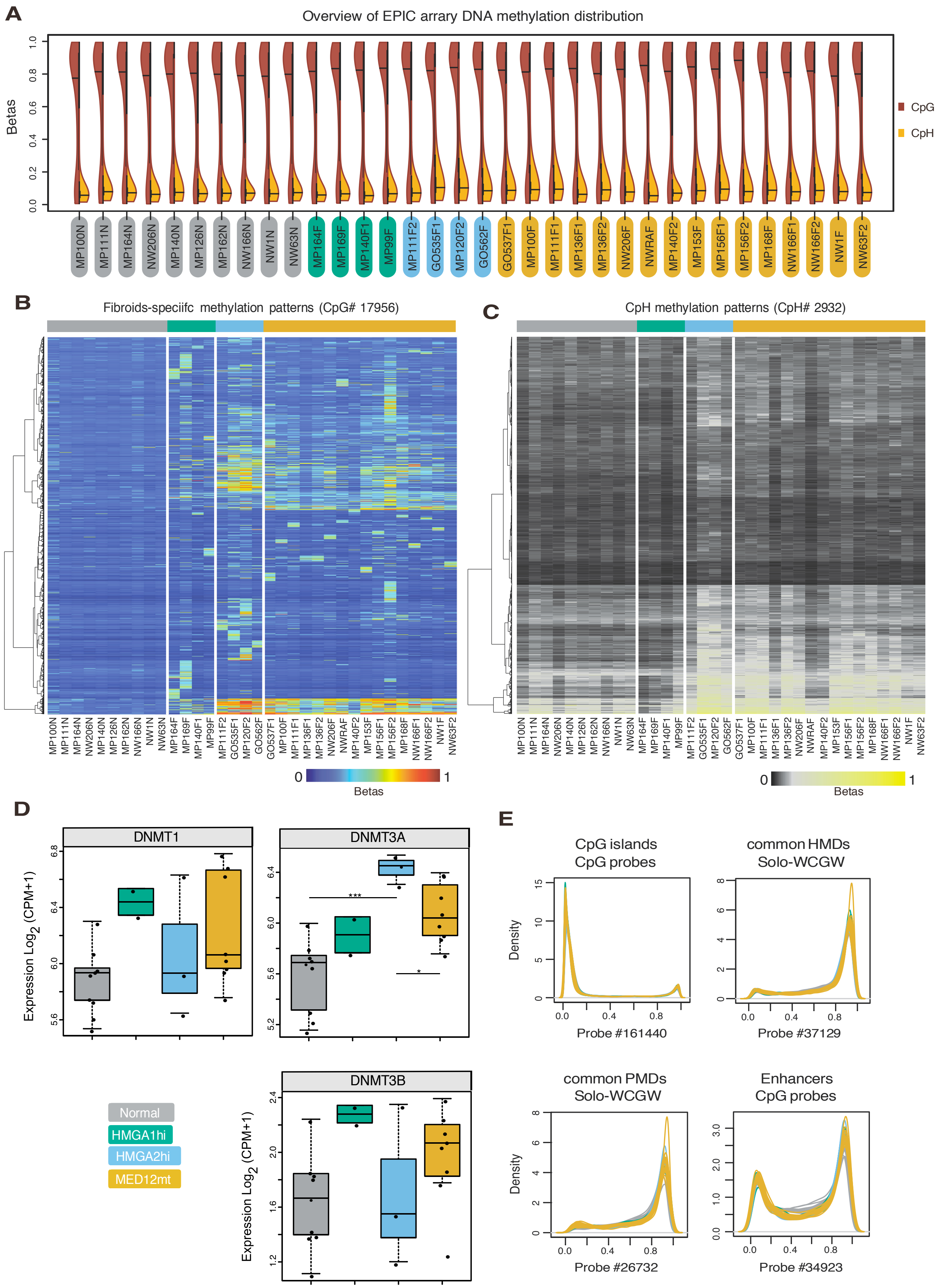

Figure S3. Methylome overview, Related to Figure 1. (A) Split bean plot of CpG (red) and CpH (orange) methylation for normal myometria (n=10), *MED12*mt (n=16), *HMGA2*hi (n=4), and *HMGA1*hi (n=4) fibroids. **(B)** Heatmap of fibroid-specific CpG methylation patterns with samples from panel A. **(C)** Heatmap of all CpH methylation with samples from panel A. **(D)** Expression levels of the indicated DNA methyltransferase (DNMT) genes from RNA-seq data comparing normal myometrium (n=9) and each of the *MED12*mt (n=8), *HMGA2*hi (n=3), *HMGA1*hi (n=2) fibroids shown as boxplots (boxes, 25–75%; whiskers, 10–90%; lines, median). Significance was determined by Wilcoxon test; *$*p<0.05$ and *** $p<0.001$.* **(E)** Sample-based distribution of CpG methylation within different genomic features: CpG islands, common HMDs (highly methylated domains), common PMDs (partially methylated domains), and enhancers. Lines are colored by methylation clusters.

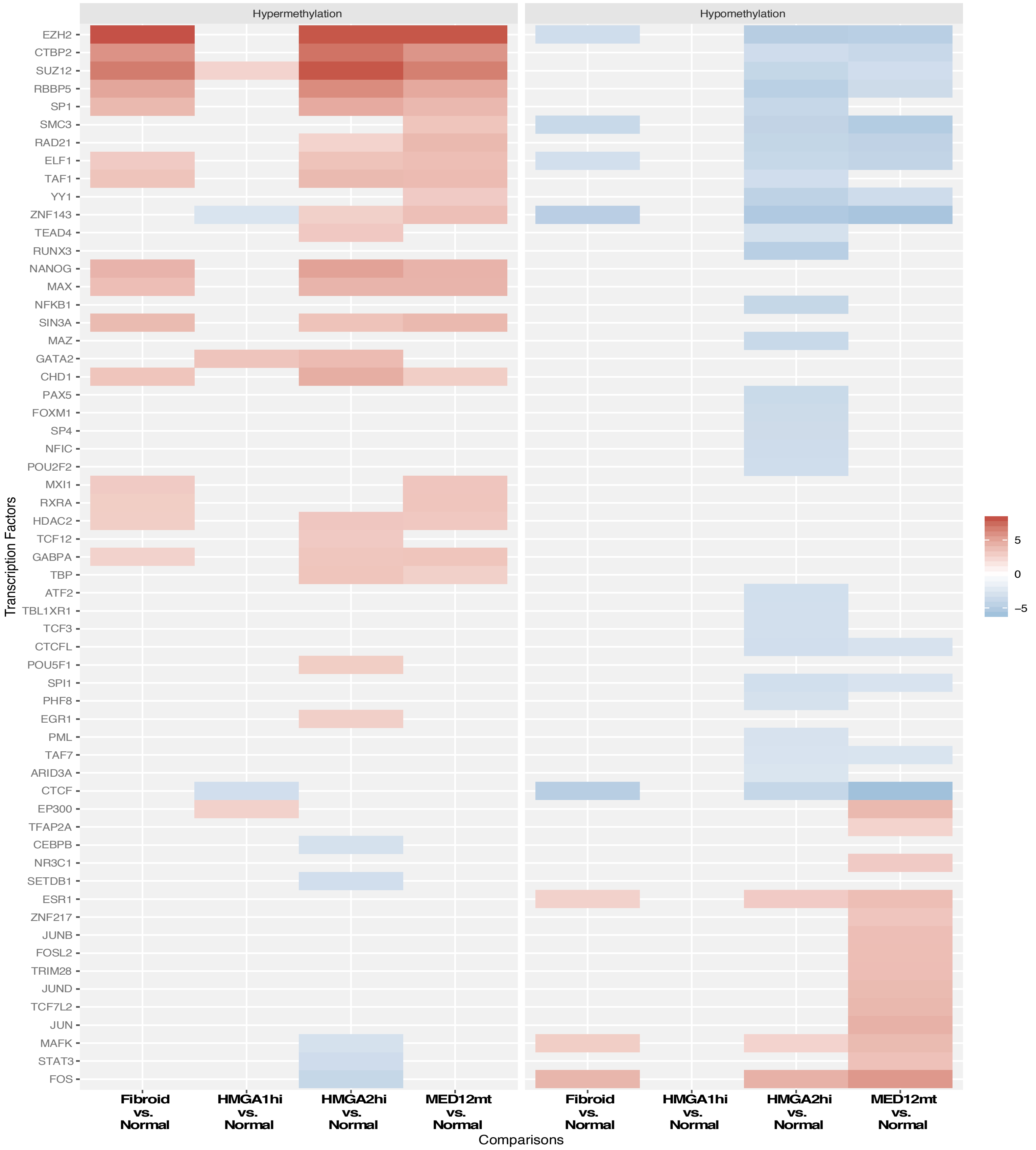

Figure S4. Enrichment or depletion analyses of TFBSs among differential methylation loci at distal elements, Related to Figure 1. Significant TFs were plotted (y axis) for each comparison (x axis) in normal myometria (n=10), MED12mt (n=16), HMGA2hi (n=4), and HMGA1hi (n=4) fibroids. Analyses were done for both hyper- and hypo-methylated loci, as indicated. Negative log10-transformed FDR values are log-scaled, and then colored as gradient red for enrichment and gradient blue for depletion.

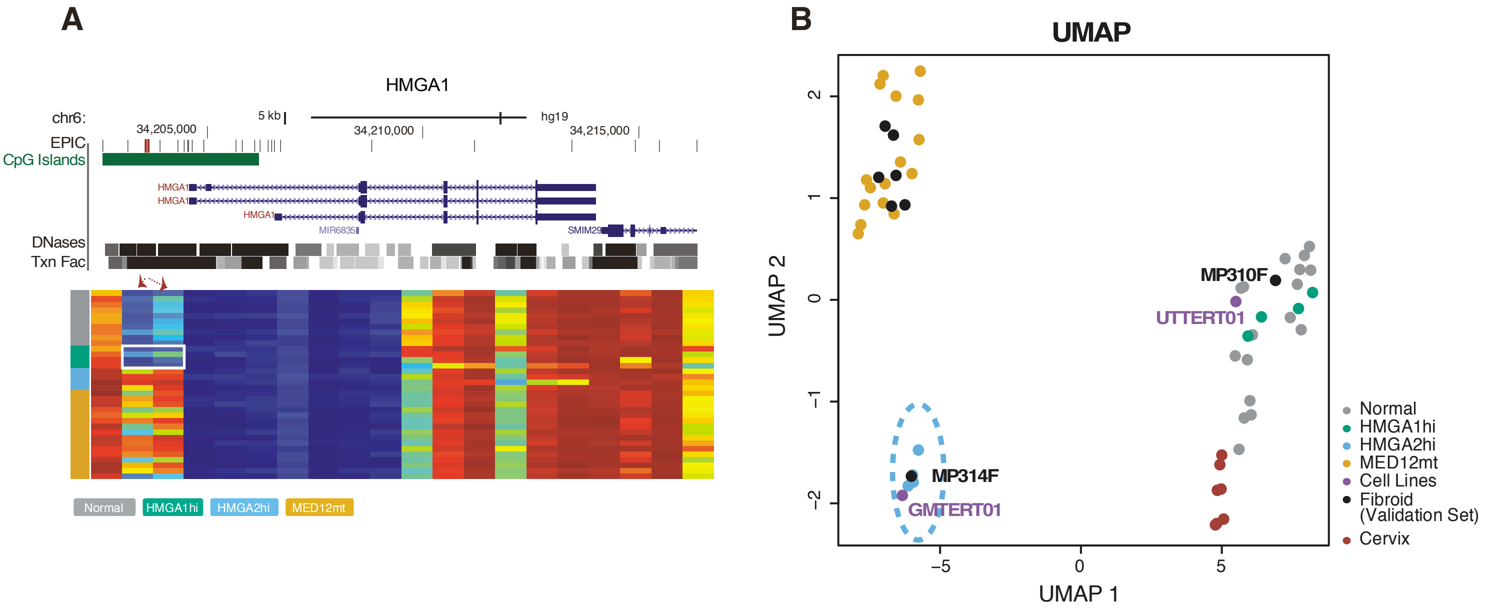

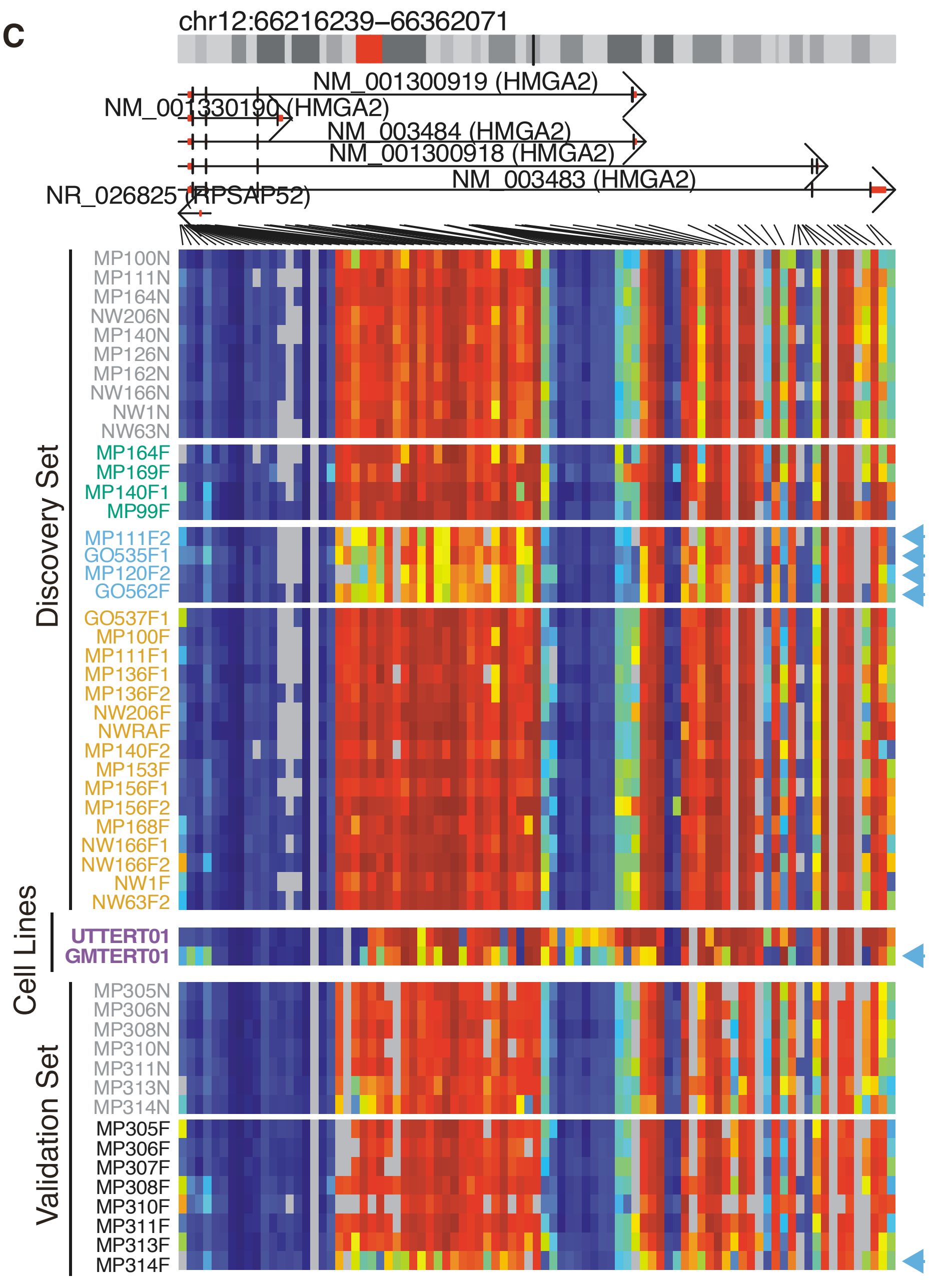

Figure S5. *HMGA1/2* Methylation, Related to Figure 3*.* (A) Samples are colored by methylation clusters, with annotation tracks of EPIC probes, *HMGA1* UCSC gene, DNases peak and transcription factor binding on top of the heatmap. Two red dashed arrows together with the white box indicate the promoter hypomethylation of normal myometria (n=10) and *HMGA1*mt (n=4) compared to other fibroids subtypes, *MED12*mt (n=16) and *HMGA2*hi (n=4). **(B)** UMAP plot based on methylation measured by EPIC array. Each dot represents one sample, colored by fibroid subtypes *MED12*mt (n=22), *HMGA1*hi (n=4), *HMGA2*hi (n=5), normal myometria (n=17), cervical stroma (n=6), and cell line category (GMTERT01, an *HMGA2* translocation fibroid cell line, and UTTERT01, a myometrial cell line (Carney et al., 2002). Black dots are fibroids from validation set. **(C)** DNA methylation pattern of fibroid signature *HMGA2* gene in discovery set, two cell lines and validation set samples in panel B. Blue arrow heads indicate *HMGA2*hi samples.

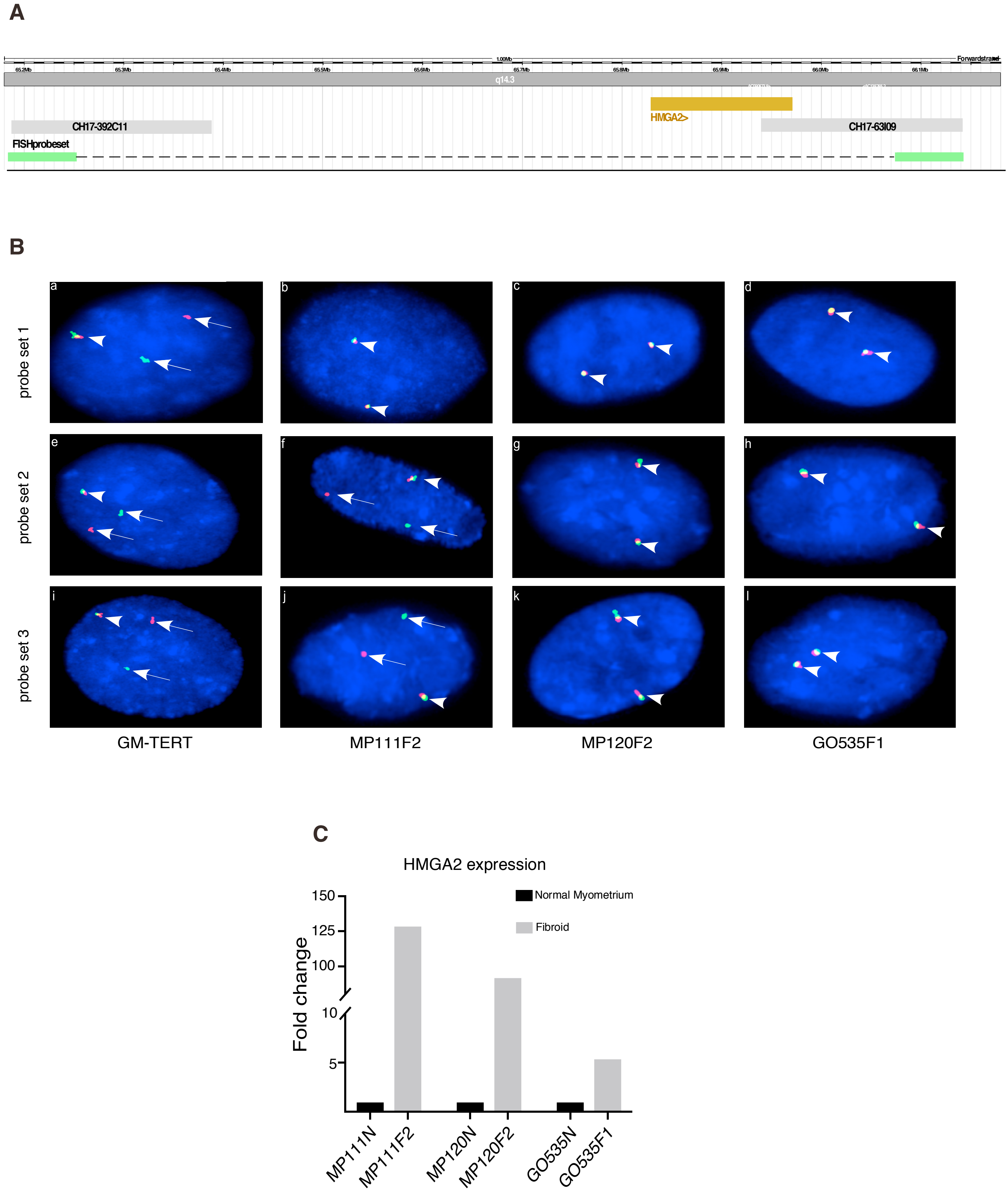

Figure S6. Mapping of *HMGA2* translocations by fluorescence in situ hybridization (FISH), Related to Figure 3. **(A)** Map of BAC probes used in FISH experiment according to UCSC GRCh38/hg38. Green bars indicate length of each individual FISH probe.**(B)** Representative nuclei, stained with DAPI in blue show fluorescent puncta using individual BAC probes, on GM-TERT (positive control) and fibroids, MP111F2, MP120F2, and GO535F1. Arrowheads indicate intact *HMGA2*, while arrows indicate *HMGA2* translocation. **(C)** qRT-PCR of *HMGA2* mRNA expression in MP111F2, MP120F2, and GO535F1 fibroids compared to corresponding normal myometrium. Results are normalized to myometria and shown as fold change.

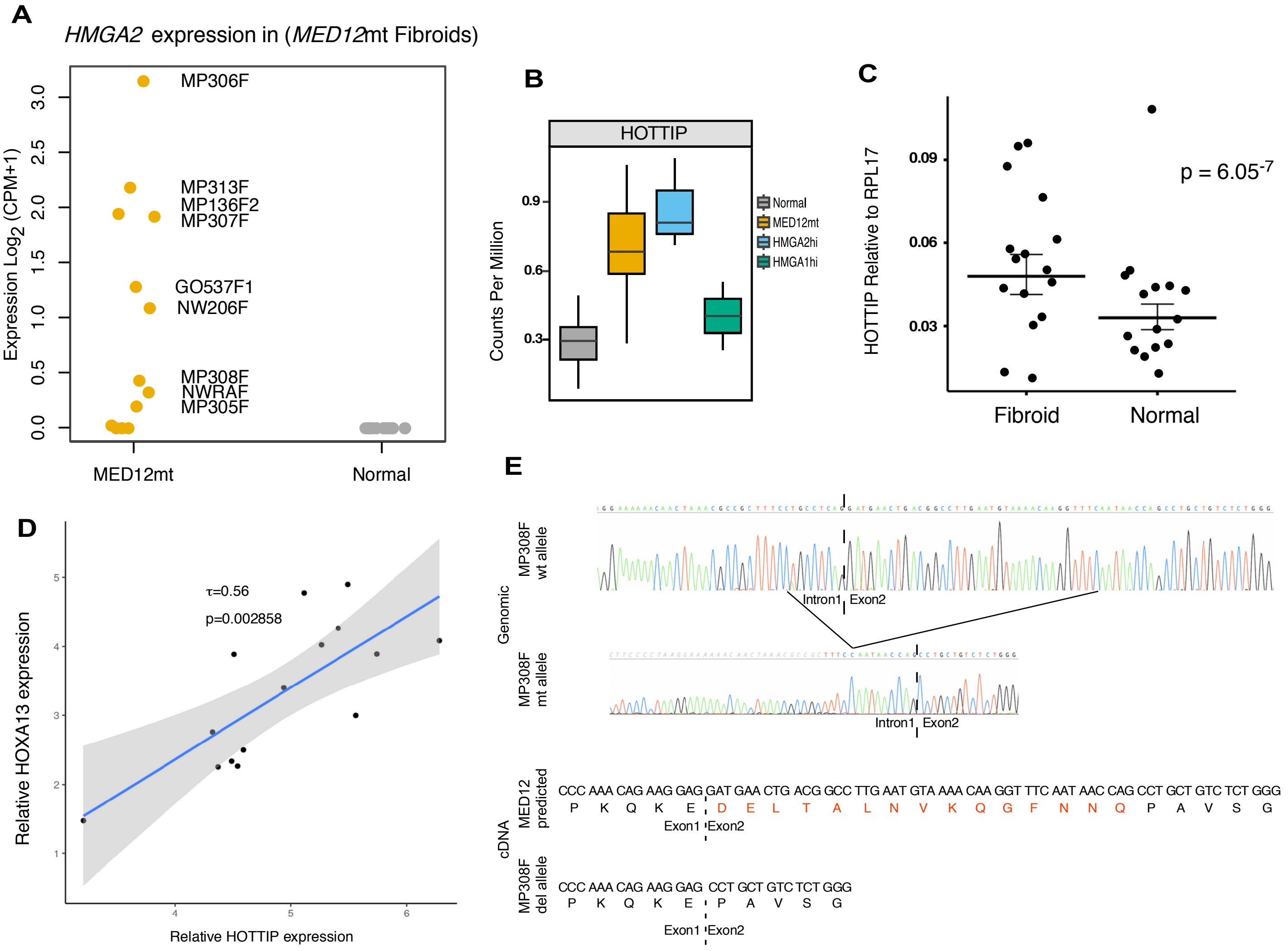

Figure S7. *HMGA2* expression in *MED12*mt subtype, *HOTTIP* expression and MP308F MED12 deletion, Related to Figure 6. **(A)** Expression levels from RNA-seq results of *HMGA2* in *MED12*mt fibroids (n=14) and normal myometrial (n=15) samples. Only those *MED12*mt fibroids with higher than normal expression of *HMGA2* are labeled. **(B)** Boxplots (boxes, 25–75%; whiskers, 10–90%; lines, median) of *HOTTIP* mRNA expression in normal myometria (n=9) compared to *MED12*mt (n=8), *HMGA2*hi (n=3), *HMGA1*hi (n=2) fibroids from the RNA-seq results. **(C)** Relative expression of *HOTTIP* by qRT-PCR compared to RPL17 housekeeping gene (y axis) between normal myometrium (n=17) and unsubtyped fibroids (n=19) in a validation set of samples. 25%-75% quartiles and median expression are indicated as horizontal lines. **(D)** Expression correlation between *HOXA13* and *HOTTIP* in the validation set of uterine fibroids(n=15). Line represents linear regression and shaded area represents 95% confidence interval for linear prediction. **(E)** Sanger sequencing of genomic *MED12* alleles of MP308F shows a 44 bp deletion that encompasses the wildtype exon 2 splice acceptor site. New splice acceptor is shown in deleted allele. *MED12* cDNA sequence and predicted amino acid sequences are shown for both the wildtype allele and the deleted allele.
